# Synthesis and Biological Evaluation of Honokiol Derivatives Bearing 3-((5-phenyl-1,3,4-oxadiazol-2-yl)methyl)oxazol-2(3H)-ones as Potential Viral Entry Inhibitors against SARS-CoV-2

**DOI:** 10.3390/ph14090885

**Published:** 2021-08-31

**Authors:** Yong Guo, Jie-Ru Meng, Jia-Zheng Liu, Ting Xu, Zhi-Yuan Zheng, Zhi-Hong Jiang, Li-Ping Bai

**Affiliations:** 1 State Key Laboratory of Quality Research in Chinese Medicine, Macau Institute for Applied Research in Medicine and Health, Guangdong-Hong Kong-Macao Joint Laboratory of Respiratory Infectious Disease, Macau University of Science and Technology, Taipa, Macau 999078, China; yguo@must.edu.mo (Y.G.); 1909853gct20002@student.must.edu.mo (J.-R.M.); 2009853DCT20001@student.must.edu.mo (J.-Z.L.); 1909853ect30003@student.must.edu.mo (T.X.); 1709853ect30001@student.must.edu.mo (Z.-Y.Z.); zhjiang@must.edu.mo (Z.-H.J.); 2 School of Pharmaceutical Sciences, Zhengzhou University, Zhengzhou 450001, China

**Keywords:** honokiol analogues, viral entry inhibitors, SARS-CoV-2, COVID-19, ACE2 blocker

## Abstract

The 2019 coronavirus disease (COVID-19) caused by SARS-CoV-2 virus infection has posed a serious danger to global health and the economy. However, SARS-CoV-2 medications that are specific and effective are still being developed. Honokiol is a bioactive component from *Magnoliae officinalis* Cortex with damp-drying effect. To develop new potent antiviral molecules, a series of novel honokiol analogues were synthesized by introducing various 3-((5-phenyl-1,3,4-oxadiazol-2-yl)methyl)oxazol-2(3*H*)-ones to its molecule. In a SARS-CoV-2 pseudovirus model, all honokiol derivatives were examined for their antiviral entry activities. As a result, **6a** and **6p** demonstrated antiviral entry effect with IC_50_ values of 29.23 and 9.82 µM, respectively. However, the parental honokiol had a very weak antiviral activity with an IC_50_ value more than 50 µM. A biolayer interfero-metry (BLI) binding assay and molecular docking study revealed that **6p** binds to human ACE2 protein with higher binding affinity and lower binding energy than the parental honokiol. A competitive ELISA assay confirmed the inhibitory effect of **6p** on SARS-CoV-2 spike RBD’s binding with ACE2. Importantly, **6a** and **6p** (TC_50_ > 100 μM) also had higher biological safety for host cells than honokiol (TC_50_ of 48.23 μM). This research may contribute to the discovery of potential viral entrance inhibitors for the SARS-CoV-2 virus, although **6p**’s antiviral efficacy needs to be validated on SARS-CoV-2 viral strains in a biosafety level 3 facility.

## 1. Introduction

As of June 2021, more than 170 million individuals had been infected worldwide since the outbreak of a novel coronavirus-associated acute respiratory disease (COVID-19) in December of 2019. Nowadays there are still no specific medications for COVID-19 patients, except for symptomatic treatment. SARS-CoV-2 necessitates the creation of specific and effective medications. ACE2, a receptor located on the outer surface of a wide variety of host cells, is the primary target to which the SARS-CoV-2 spike attaches. ACE2-targeted molecules, such as oroxylin A [1], peptide AYp28 [2], doxepin [3], and dexamethasone [4], have been reported to block SARS-CoV-2 spike RBD’s interactions with ACE2, and thus exert antiviral entry effects on SARS-CoV-2. Therefore, targeting ACE2 with organic molecules is an alternative strategy for the discovery of effective antiviral drugs to prevent or combat SARS-CoV-2. *Magnoliae officinalis* Cortex (also known as “Hou Po” in Chinese) was a traditional Chinese medicine (TCM) with a damp-drying effect. It was a major herb in a number of TCM prescriptions or Chinese patent medications, including Hua-Shi-Bai-Du formulation [5], Dayuan Decoction [6], and Huoxiang Zhengqi Dropping Pills [7] which were useful and effective in treating COVID-19 in clinics. As a main and bioactive component in this TCM, honokiol has been shown to prevent viral entry into host cells, hence fighting viruses including hepatitis C virus (HCV) [8] and dengue fever virus (DENV) [9]. Therefore, honokiol was considered as an ideal molecule for structural modification in order to find new antiviral entry inhibitors for SARS-CoV-2.

An ideal antiviral molecule is required to possess low cytotoxicity to host cells and high antiviral efficacy. In our previous studies [10,11], it was found that honokiol was toxic to both cancerous and non-cancer cells, probably due to the presence of two phenolic hydroxyl groups. The introduction of 1,3,4-oxadiazole-linked phenyls to honokiol (**9a**–**9o** in the literature [10]) could reduce cytotoxic effects on non-cancer cells. Additionally, compounds containing 1,3,4-oxadiazole moiety have recently been reported to exhibit good antiviral activity against SARS-CoV-2 [12,13]. Our preliminary computational study also exhibited that the oxadiazole linker could form conventional hydrogen bonding interactions with active sites in ACE2 protein. Based on the above considerations, 1,3,4-oxadiazole-linked phenyls were employed to modify the molecule of honokiol with the design of oxazolone group to block phenolic hydroxyl groups. Herein, a series of honokiol analogues were synthesized by introducing various 3-((5-phenyl-1,3,4-oxadiazol-2-yl)methyl)oxazol-2(3*H*)-ones into its molecule. Their antiviral entrance properties were tested in a SARS-CoV-2 pseudovirus model based on the viral spike protein by using a lentiviral system that included a luciferase reporter gene for quantifying spike and ACE2-mediated virus entry in a biosafety level 2 (BSL-2) facility [14,15,16,17,18,19,20,21]. The in vitro binding affinities of antiviral derivatives were subsequently examined with both SARS-CoV-2 spike RBD and human ACE2 proteins by BLI binding assay [22,23]. Subsequently, a molecular docking study was also employed to further predict their active binding sites in SARS-CoV-2 human ACE2 receptor. Finally, a competitive ELISA assay was carried out to investigate the inhibitory effect of antiviral analogues on the binding interaction of spike RBD with ACE2. This research could lead to potential SARS-CoV-2 viral entrance inhibitors.

## 2. Results and Discussion

### 2.1. Chemistry

**Scheme 1:**
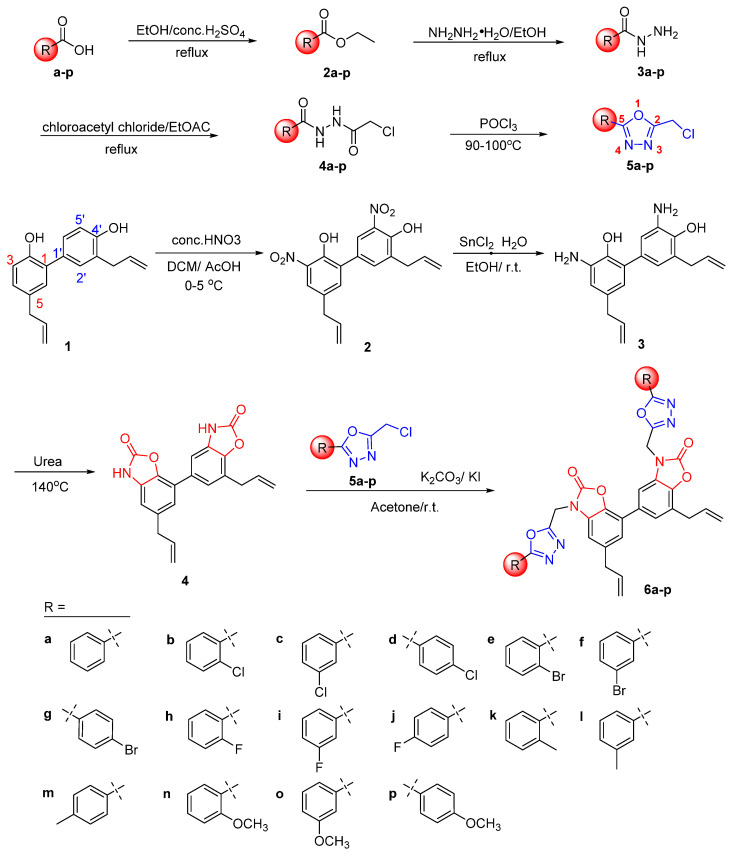
Synthetic routes of honokiol derivatives **6a**-**p** bearing 3-((5-phenyl-1,3,4-oxadiazol-2-yl)methyl)oxazol-2(3H)-ones.

As shown in Scheme 1, various substituted benzoic acids (**a**-**p**) were used as starting materials to firstly prepare key intermediates 2-chloromethyl-5-substituted phenyl-1,3,4-oxadiazoles **5a**-**p** through four steps of esterification, hydrazinolysis, amidation, and cyclization according to the reported procedures [11]. Secondly, the nitro group was successfully introduced at the *ortho* position of hydroxyl groups of the honokiol *via* nitration reaction to obtain 3,5′-dinitrohonokiol (**2**), which was then reduced by SnCl_2_ to produce 3,5′-diaminohonokiol (**3**). Subsequently, 3,5′-diaminohonokiol (**3**) was reacted with urea at 140 °C to obtain another key cyclized product **4** [24]. Finally, a series of novel honokiol derivatives **6a**-**p** containing various 3-((5-phenyl-1,3,4-oxadiazol-2-yl)methyl)oxazol-2(3*H*)-ones were smoothly prepared by reacting compound **4** with the corresponding 2-chloromethyl-5-substituted phenyl-1,3,4-oxadiazoles **5a**-**p** in the presence of K_2_CO_3_ and KI [25]. All honokiol derivatives **6a**-**p** were characterized by IR, HRMS and ^1^H/^13^C NMR. The purities of analogues **6a-p** were determined more than 95% by HPLC analysis before a biological evaluation.

### 2.2. Biological Evaluation

#### 2.2.1. Antiviral Activities of Honokiol Analogues **6a**-**p** against the Entry of SARS-CoV-2 Pseudovirus into Host Cells

**Figure 1:**
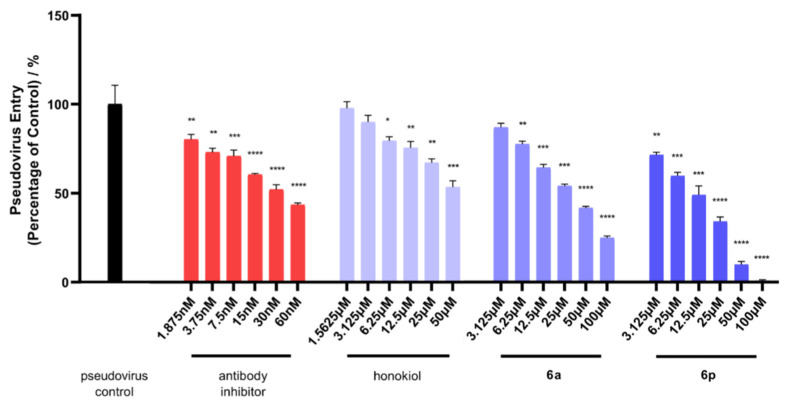
Effect of honokiol, representative **6a** and **6p** on the entrance of SARS-CoV-2 pseudovirus into HEK-293T-ACE2^h^ cells. The luciferase luminescence value of the pseudovirus control was defined as 100%. The values of luminescence of either antibody inhibitor-treated or compound-treated groups were normalized accordingly. Results were expressed as mean ± standard error (n = 4). * *p* < 0.05, ** *p* < 0.01, *** *p* < 0.001, **** *p* < 0.0001 compared with pseudovirus control group. The experiments were repeated at least three times.

In our BSL-2 facility, SARS-CoV-2 spike pseudotyped viruses with luciferase reporter were constructed to infect HEK-293T-ACE2^h^ cells that produce high levels of human ACE2 (angiotensin-converting enzyme 2). This experimental model could help researchers find potential antiviral compounds that prevent SARS-CoV-2 from infecting host cells overexpressing ACE2 receptor. Initially, the maximum nontoxic concentration (CC_0_) values of honokiol derivatives **6a**-**p** were determined against the host cell of HEK-293T-ACE2^h^ by MTT (Table 1). Subsequently, the antiviral entry activities of compounds were evaluated under their doses of no higher than CC_0_ values. HEK-293T-ACE2^h^ cells infected only with SARS-CoV-2 spike pseudovirus were considered as controls, and the value of luciferase luminescence of the control was defined as 100%. A SARS-CoV-2 antibody was utilized as a positive viral entry inhibitor. As shown in Figure 1 and Table 1, the positive inhibitor suppressed the entry of the pseudovirus with an IC_50_ value of 0.035 µM. Compounds **6a**, **6h,** and **6p** demonstrated IC_50_ values of 29.23, 61.58, and 9.82 µM against the pseudovirus entry, respectively. However, the parental honokiol only exhibited very weak inhibitory effect on pseudovirus entry with IC_50_ value more than 50 µM. The IC_50_ values of all the other analogues **6b**-**g**, **6i**-**o** were all more than their CC_0_ values. With TC_50_ values greater than 100 µM, **6a**, **6h**, and **6p** had a less cytotoxic effect on HEK-293T-ACE2^h^ host cells than honokiol (TC_50_ of 48.23 µM). Taken together, **6p** had the most potent antiviral entry effect on the pseudovirus with a selectivity index (SI) higher than 10.18 among all honokiol derivatives.

#### 2.2.2. Biolayer Interferometry (BLI) Binding Assay

**Figure 2:**
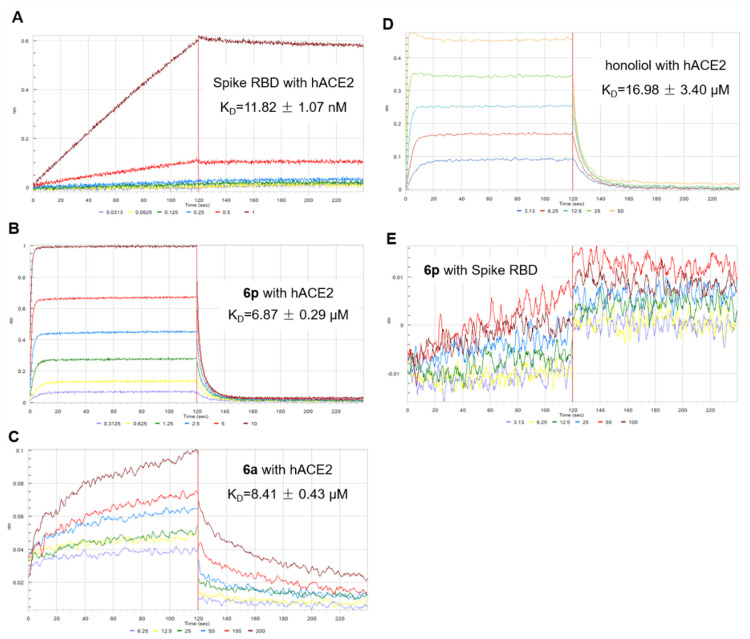
Binding curves of **6p** and **6a** with human ACE2 and SARS-CoV-2 spike RBD proteins. (**A**) The interaction of SARS-CoV-2 spike RBD (0.03125, 0.0625, 0.125, 0.25, 0.5 and 1 nM) with human ACE2 protein. (**B**) Compound **6p** (0.3125, 0.625, 1.25, 2.5, 5 and 10 µM) binding to human ACE2 protein. (**C**) Compound **6a** (6.25, 12.5, 25, 50, 100 and 200 µM) binding to human ACE2 protein. (**D**) Honokiol (3.125, 6.25, 12.5, 25 and 50 µM) binding to human ACE2 protein. (**E**) The interaction of **6p** (3.125, 6.25, 12.5, 25, 50 and 100 µM) with spike RBD protein.

The attachment of the spike receptor binding domain (RBD) to the ACE2 receptor is the first key and critical step in the SARS-CoV-2 virus infecting its host cells. The binding behaviors of honokiol and its analogue **6p** with both SARS-CoV-2 spike RBD and human ACE2 protein were thus explored by a BLI binding assay. As illustrated in Figure 2 and Table 2, SARS-CoV-2 spike RBD binds to human ACE2 with an equilibrium dissociation constant (K_D_) value of 11.82 nM. Compound **6p** binds to ACE2 protein with K_D_ value of 6.87 µM, while it doesn’t show interaction with spike RBD even in the tested concentration up to 100 µM. Compound **6a** had a K_D_ value of 8.41 µM with ACE2 protein. Moreover, the binding ability of both **6p** and **6a** with human ACE2 protein was higher than that of honokiol (K_D_ value of 16.98 µM). The finding revealed that **6p** and **6a** might effectively prevent SARS-CoV-2 from binding to the host’s ACE2 receptor by blocking ACE2 receptor of host cells.

#### 2.2.3. Molecular Modeling Study

**Figure 3:**
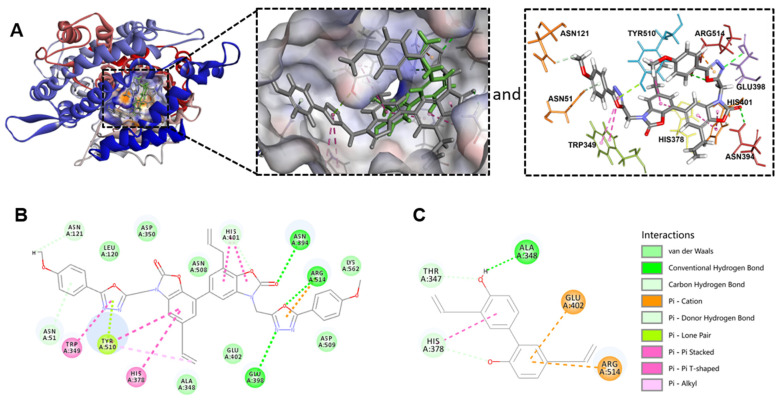
Docking simulation of **6p** and honokiol in the active sites of human ACE2 receptor (PDB code: 1R4L). (**A**) 3D binding models of **6p** (gray) and honokiol (green) with human ACE2. (**B**) 2D binding model of **6p** with human ACE2. (**C**) 2D binding model of honokiol with human ACE2.

CDOCKER is an approach for producing high-precision molecular docking results under the CHARMm force field [26]. Therefore, CDOCKER of Discovery Studio software was carried out to predict the binding of **6p**, **6a** and honokiol with human ACE2 receptor (Figure 3). The data in Figure 3A showed that **6p** interacts with ACE2 protein via Tyr510 and Arg514 which were the same binding sites as that of a reported ACE2 inhibitor [27]. Moreover, **6p** also formed other interactions with human ACE2 protein including hydrogen bond with Asn394 and Glu398 (Figure 3A,B). A more negative binding energy indicated a more stable interaction between the compound and the protein. As reflected from the calculated binding energy of **6p** (−65.339 kcal/mol), **6a** (−59.849 kcal/mol) and honokiol (−31.963 kcal/mol), the binding ability of **6p** and **6a** with ACE2 is much stronger than that of honokiol (Table 2). Taken together, these data suggested that introduction of 3-((5-phenyl-1,3,4-oxadiazol-2-yl)methyl)oxazol-2(3*H*)-ones in honokiol’s molecule enhanced binding interaction of **6p** and **6a** with human ACE2 receptor.

#### 2.2.4. Inhibitory Effect of **6p** on Binding of SARS-CoV-2 Spike RBD with ACE2 Protein

**Figure 4:**
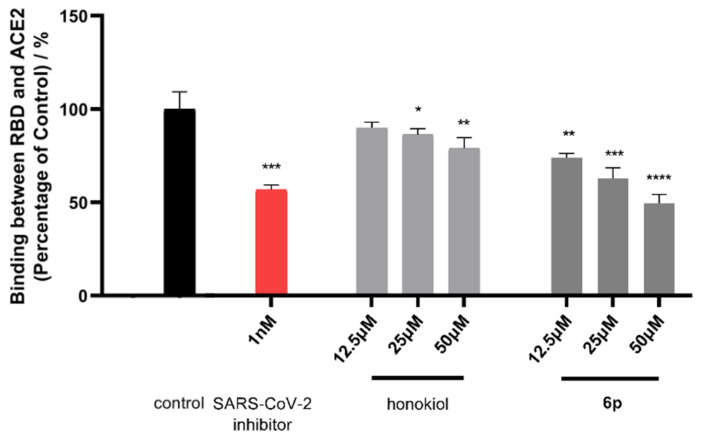
Binding activity of SARS-CoV-2 spike RBD with ACE2 protein in the absence (control) and presence of honokiol and **6p** (12.5, 25 and 50 µM) by a competitive ELISA assay. A SARS-CoV-2 antibody inhibitor (1 nM) was utilized as a positive control. The OD value of the control group was defined as 100%. The OD values of either antibody inhibitor-treated or compound-treated groups were normalized accordingly. Results were expressed as mean ± standard error (n = 3). * *p* < 0.05, ** *p* < 0.01, *** *p* < 0.001, **** *p* < 0.0001 compared with the control group. The experiments were repeated three times.

A competitive ELISA assay was conducted to further investigate the interventive effect of compound **6p** on the binding of SARS-CoV-2 spike RBD with ACE2 protein. A SARS-CoV-2 antibody inhibitor (IC_50_ value of 2.59 nM) was employed as a positive control in this assay. As a result (Figure 4), **6p** can suppress the binding of spike RBD and ACE2 with inhibitory rate of 26.08%, 37.16%, and 50.41% under tested concentration of 12.5, 25, and 50 µM, respectively. Honokiol also showed a relatively weak inhibitory rate of 13.81% and 20.94% under 25 and 50 µM, respectively. In conjunction with the BLI and molecular simulation results, the dose-dependent inhibitory effect of **6p** on spike RBD’s binding with ACE2 provided supporting evidence that **6p** occupied the active sites of ACE2 to prevent the attachment of spike RBD.

#### 2.2.5. Structure-Activity Relationship Analysis

Based on the above findings, an analysis of structure-activity relationship (SAR) was concluded as follows. Firstly, 3-((5-phenyl-1,3,4-oxadiazol-2-yl)methyl)oxazol-2(3*H*)-one was an indispensable functional group for the antiviral entry activity of **6a**. The introduction of this functional group to honokiol resulted in an IC_50_ value of 29.23 µM against entry of SARS-CoV-2 spike pseudotyped virus into host cells, a potent binding affinity (K_D_ value of 8.41 µM) with ACE2 protein, and a low binding energy (−59.849 kcal/mol) with ACE2 protein when compared with that of the parental honokiol. Moreover, the cytotoxicity of **6a** (TC_50_ > 100 µM) was also much lower than that (TC_50_ of 48.23 µM) of the parental honokiol against HEK-293T-ACE2^h^ cells. Secondly, an electron-donating methoxyl substituent at the *para*-position of **6a** was more beneficial to enhance the antiviral entry activity of **6p** due to the fact that **6p** (IC_50_ of 9.82 μM) exhibited 2.97 times greater inhibitory activity against pseudovirus entry and a more potent binding affinity (KD value of 6.87 µM) with ACE2 protein than **6a**. The importance of methoxyl at the *para*-position of **6a** was also supported by the evidence that **6p** had a more stable interaction (binding energy −65.339 kcal/mol) with ACE2 protein than **6a** probably due to the additional carbon hydrogen bonding interaction of one methoxyl with the Asn121 site in ACE2 (Figure 3A,B). Thirdly, the electron-withdrawing –F group at *ortho*-position of **6a** led to a 2.10-fold loss in antiviral entry activity of **6h** (IC_50_ of 61.58 μM), while the presence of –F at *meta*- and *para*-positions of **6a** resulted in complete loss of antiviral activities of both **6i** and **6j**. It was postulated that the presence of –F group may reduce the binding stability of **6a** with ACE2. Finally, other electron-withdrawing –Cl, –Br, and electron-donating methyl substituents at any position of **6a** also led to complete loss of antiviral entry activities.

## 3. Materials and Methods

### 3.1. Reagents and Apparatus

Honokiol was obtained from Xianding Biological Technology Co., Ltd. (Shanghai, China). Various substituted benzoic acids, chloroacetyl chloride, stannous chloride, and phosphorus oxychloride were purchased from J & K Scientific Ltd. and used without further purification. Melting points (m.p.) of all honokiol derivatives were measured by a digital melting-point apparatus (XT-4, Beijing, China) that was not corrected. Infrared spectra (IR) were determined by a FT-IR spectrometer (PE-1710, Waltham, MA, USA). A Bruker 400/100 MHz NMR spectrometer (Avance, Bremerhaven, Germany) was used to measure ^1^H and ^13^C NMR spectra of honokiol derivatives **6a**-**p**. High resolution mass spectra (HRMS) were measured on an Agilent 6545 ESI-QTOF-MS instrument (Agilent Technologies, Santa Clara, CA, USA). The purities of all synthetic derivatives were determined on an Agilent 1200 HPLC-DAD apparatus.

### 3.2. Synthesis of 2-chloromethyl-5-substituted phenyl-1,3,4-oxadiazoles **5a**-**p**, and Compounds **2**-**4**

To a solution of substituted benzoic acids **a**-**p** (10 mmol) in EtOH (15 mL), concentrated sulfuric acid (0.1 mL) was added, and the reaction solution was refluxed for 24 h. Then the solution was evaporated under reduced pressure, and the mixture was purified by silica gel column chromatography (CC) to afford compounds **2a**-**p**. Subsequently, compounds **2a**-**p** were reacted with hydrazine hydrate (15 mmol) in EtOH under reflux to obtain various substituted benzoyl hydrazines **3a**-**p**. Chloroacetylation of **3a**-**p** was performed with chloroacetyl chloride (10 mmol) in EtOAc under reflux condition to afford compounds **4a**-**p**. Finally, compounds **4a**-**p** were treated with POCl_3_ at 90–100 °C to successfully obtain the key intermediates of 2-chloromethyl-5-substituted phenyl-1,3,4-oxadiazoles **5a**-**p**.

To a solution of honokiol (37.5 mmol) in acetic acid (10 mL), concentrated nitric acid (112.7 mmol) was slowly added. When the reaction was completed, the mixture was poured into ice water and adjusted to pH = 7 with 5% sodium bicarbonate solution, then extracted with dichloromethane (20 mL × 3). The organic layer was dried with anhydrous sodium sulfate, concentrated under reduced pressure, and purified by silica gel CC to obtain 3,5′-dinitrohonokiol (**2**). A mixture of compound **2** (30 mmol) and SnCl_2_ (90 mmol) was dissolved in EtOH, and stirred at 25 °C. After the reaction was completed, the reaction solution was concentrated *in vacuo*, then the residue was diluted by EtOAc and adjusted to pH = 7 with 5% sodium bicarbonate solution. Finally, the EtOAc solution was dried over anhydrous Na_2_SO_4_, concentrated by a rotary evaporator, and purified by silica gel CC to give the product of 3,5′-diaminohonokiol (**3**). A mixture of compound **3** (20 mmol) and urea (80 mmol) was stirred at 140 °C for about 12 h. When the reaction was completed according to the TLC analysis, 10% hydrochloric acid solution was added to the reaction mixture, and then the mixture was extracted with ethyl acetate (20 mL × 3). Subsequently, the organic layer was dried over Na_2_SO_4_, concentrated under reduced pressure, and purified by silica gel CC to obtain cyclized product **4**.

### 3.3. Synthesis of Compounds **6a**-**p**

To a solution of compound **4** (0.5 mmol) in acetone (5 mL), K_2_CO_3_ (1.5 mmol), KI (0.1 mmol), and the corresponding 2-chloromethyl-5-substituted phenyl-1,3,4-oxadiazoles **5a**-**p** (0.6 mmol) were added, and then the reaction mixture was stirred at room temperature. When the reaction was completed, the mixture was filtered, concentrated under reduced pressure, and finally purified by PTLC to obtain the target compounds **6a**-**p**. The structural identification data of compound **6a-p** (Figures S1–S47) were illustrated as follows.

**Data for 6a**: White solid, yield: 85%, m.p. 202–205 °C; IR cm^−1^(KBr): 1776, 1446, 1342, 1067, 707, 690; ^1^H NMR (400 MHz CDCl_3_) *δ*: 7.99–8.03 (m, 4H, -Ph), 7.47–7.54 (m, 6H, -Ph), 7.31 (s, 2H, -Ph), 7.04 (s, 1H, -Ph), 6.97 (d, *J* = 1.2 Hz, 1H, -Ph), 5.90–6.00 (m, 2H, -C*H*=CH_2_), 5.38 (s, 2H, -C*H*_2_-N), 5.34 (s, 2H, -C*H*_2_-N), 5.09–5.20 (m, 4H, -CH=C*H_2_*), 3.56 (d, *J* = 6.8 Hz, 2H, -C*H*_2_-CH=CH_2_ ), 3.43 (d, *J* = 6.4 Hz, 2H, -C*H*_2_-CH=CH_2_); ^13^C NMR (100 MHz CDCl_3_) *δ*: 166.13, 160.23, 153.97, 140.80, 138.03, 137.39, 136.56, 134.39, 132.29, 132.24, 131.22, 130.53, 130.14, 129.14, 129.11, 127.18, 124.61, 123.62, 123.39, 123.11, 117.36, 116.82, 108.23, 106.89, 40.07, 36.88, 33.33; HRMS: calcd for C_38_H_29_N_6_O_6_ ([M+H]^+^) 665.2070, found 665.2145. 

**Data for 6b**: White solid, yield: 70%, m.p. 167–170 °C; IR cm^−1^(KBr): 2923, 1776, 1581, 1442, 1342, 731; ^1^H NMR (400 MHz CDCl_3_) *δ*: 7.93–7.97 (m, 2H, -Ph), 7.31–7.52 (m, 8H, -Ph), 7.05 (d, *J* = 1.2 Hz, 1H, -Ph), 6.97 (d, *J* = 1.2 Hz, 1H, -Ph), 5.90–6.00 (m, 2H, -C*H*=CH_2_), 5.41 (s, 2H, -C*H*_2_-N), 5.37 (s, 2H, -C*H*_2_-N), 5.09–5.20 (m, 4H, -CH=C*H_2_*), 3.56 (d, *J* = 6.8 Hz, 2H, -C*H*_2_-CH=CH_2_), 3.44 (d, *J* = 6.8 Hz, 2H, -C*H*_2_-CH=CH_2_); ^13^C NMR (100 MHz CDCl_3_) *δ*: 164.42, 164.38, 160.66, 160.65, 153.91, 140.81, 138.03, 137.35, 136.55, 134.38, 133.35, 133.32, 132.88, 132.81, 131.34, 131.29, 131.17, 130.50, 130.12, 127.16, 127.14, 124.61, 123.60, 123.40, 122.40, 117.35, 116.84, 108.24, 106.89, 40.08, 36.93, 36.88, 33.33; HRMS: calcd for C_38_H_27_^35^Cl_2_N_6_O_6_ ([M+H]^+^) 733.1291, found 733.1363; calcd for C_38_H_27_^35^Cl ^37^ClN_6_O_6_ ([M+H]^+^) 735.1291, found 735.1346.

**Data for 6c**: White solid, yield: 93%, m.p. 155–158 °C; IR cm^−1^(KBr): 2972, 1784, 1770, 1438, 1343, 1042; ^1^H NMR (400 MHz CDCl_3_) *δ*: 7.90–8.01 (m, 4H, -Ph), 7.42–7.51 (m, 4H, -Ph), 7.31 (s, 2H, -Ph), 7.05 (d, *J* = 1.2 Hz, 1H, -Ph), 6.97 (d, *J* = 1.2 Hz, 1H, -Ph), 5.90–6.00 (m, 2H, -C*H*=CH_2_), 5.39 (s, 2H, -C*H*_2_-N), 5.34 (s, 2H, -C*H*_2_-N), 5.10–5.20 (m, 4H, -CH=C*H_2_*), 3.56 (d, *J* = 6.4 Hz, 2H, -C*H*_2_-CH=CH_2_), 3.44 (d, *J* = 6.8 Hz, 2H, -C*H*_2_-CH=CH_2_); ^13^C NMR (100 MHz CDCl_3_) *δ*: 165.03, 164.99, 160.55, 160.52, 153.91, 153.87, 140.80, 138.00, 137.43, 136.52, 135.34, 135.28, 134.34, 132.37, 132.31, 131.18, 130.50, 130.45, 130.05, 124.68, 124.62, 123.69, 123.61, 123.42, 117.40, 116.89, 108.16, 106.85, 40.07, 36.87, 36.81, 33.34; HRMS: calcd for C_38_H_27_^35^Cl_2_N_6_O_6_ ([M+H]^+^) 733.1291, found 733.1371; calcd for C_38_H_27_^35^Cl^37^ClN_6_O_6_ ([M+H]^+^) 735.1291, found 735.1354.

**Data for 6d:** White solid, yield: 66%, m.p. 190–193 °C; IR cm^−1^(KBr): 2972, 1776, 1609, 1483, 1442, 1095; ^1^H NMR (400 MHz CDCl_3_) *δ*: 7.94–7.98 (m, 4H, -Ph), 7.45–7.48 (m, 4H, -Ph), 7.30–7.32 (m, 2H, -Ph), 7.04 (d, *J* = 1.2 Hz, 1H, -Ph), 6.97 (d, *J* = 1.2 Hz, 1H, -Ph), 5.90–6.02 (m, 2H, -C*H*=CH_2_), 5.38 (s, 2H, -C*H*_2_-N), 5.33 (s, 2H, -C*H*_2_-N), 5.10-5.20 (m, 4H, -CH=C*H_2_*), 3.56 (d, *J* = 6.8 Hz, 2H, -C*H*_2_-CH=CH_2_), 3.43 (d, *J* = 6.8 Hz, 2H, -C*H*_2_-CH=CH_2_); ^13^C NMR (100 MHz CDCl_3_) *δ*: 165.36, 160.37, 153.91, 140.80, 138.71, 138.65, 137.43, 136.54, 134.35, 131.18, 130.48, 130.08, 129.57, 129.55, 128.47, 128.45, 124.58, 123.69, 123.62, 123.41, 121.55, 117.40, 116.85, 108.20, 106.88, 40.06, 36.83, 33.33; HRMS: calcd for C_38_H_27_^35^Cl_2_N_6_O_6_ ([M+H]^+^) 733.1291, found 733.1364; calcd for C_38_H_27_^35^Cl^37^ClN_6_O_6_ ([M+H]^+^) 735.1291, found 735.1344.

**Data for 6e:** White solid, yield: 57%, m.p. 147–150 °C; IR cm^−1^(KBr): 2979, 1778, 1442, 1343, 757, 727; ^1^H NMR (400 MHz CDCl_3_) *δ*: 7.88–7.90 (m, 2H, -Ph), 7.67–7.73 (m, 2H, -Ph), 7.31–7.44 (m, 6H, -Ph), 7.05 (d, *J* = 1.2 Hz, 1H, -Ph), 6.96 (d, *J* = 1.2 Hz, 1H, -Ph), 5.93–6.00 (m, 2H, -C*H*=CH_2_), 5.41 (s, 2H, -C*H*_2_-N), 5.37 (s, 2H, -C*H*_2_-N), 5.09–5.20 (m, 4H, -CH=C*H_2_*), 3.56 (d, *J* = 6.4 Hz, 2H, -C*H*_2_-CH=CH_2_), 3.44 (d, *J* = 6.8 Hz, 2H, -C*H*_2_-CH=CH_2_); ^13^C NMR (100 MHz CDCl_3_) *δ*: 164.95, 164.90, 160.71, 153.94, 140.83, 138.04, 137.35, 136.55, 134.65, 134.59, 134.38, 132.95, 132.88, 131.80, 131.76, 131.16, 130.50, 130.11, 127.67, 127.65, 124.61, 124.50, 123.61, 123.39, 121.77, 121.73, 117.36, 116.86, 108.25, 106.91, 40.08, 36.91, 33.33; HRMS: calcd for C_38_H_27_^79^Br_2_N_6_O_6_ ([M+H]^+^) 821.0281, found 821.0354; calcd for C_38_H_27_^79^Br^81^BrN_6_O_6_ ([M+H]^+^) 823.0281, found 823.0343; calcd for C_38_H_27_^81^Br^81^BrN_6_O_6_ ([M+H]^+^) 825.0281, found 825.0330.

**Data for 6f:** White solid, yield: 79%, m.p. 127–130 °C; IR cm^−1^(KBr): 3066, 2965, 1772, 1444, 1342, 722; ^1^H NMR (400 MHz CDCl_3_) *δ*: 8.15–8.17 (m, 2H, -Ph), 7.95–7.98 (m, 2H, -Ph), 7.64–7.66 (m, 2H, -Ph), 7.31–7.39 (m, 4H, -Ph), 7.05 (d, *J* = 0.8 Hz, 1H, -Ph), 6.97 (d, *J* = 1.2 Hz, 1H, -Ph), 5.91–6.03 (m, 2H, -C*H*=CH_2_), 5.39 (s, 2H, -C*H*_2_-N), 5.34 (s, 2H, -C*H*_2_-N), 5.10–5.21 (m, 4H, -CH=C*H_2_*), 3.56 (d, *J* = 6.8 Hz, 2H, -C*H*_2_-CH=CH_2_), 3.44 (d, *J* = 6.8 Hz, 2H, -C*H*_2_-CH=CH_2_); ^13^C NMR (100 MHz CDCl_3_) *δ*:164.89, 164.84, 160.56, 160.53, 153.91, 153.88, 140.80, 138.01, 137.44, 136.52, 135.29, 135.23, 134.34, 131.18, 130.71, 130.45, 130.05, 129.98, 125.71, 124.89, 124.62, 123.70, 123.61, 123.42, 123.18, 123.13, 117.40, 116.90, 108.16, 106.86, 40.07, 36.87, 36.81, 33.34; HRMS: calcd for C_38_H_27_^79^Br_2_N_6_O_6_ ([M+H]^+^) 821.0281, found 821.0349; calcd for C_38_H_27_^79^Br^81^BrN_6_O_6_ ([M+H]^+^) 823.0281, found 823.0330; calcd for C_38_H_27_^81^Br^81^BrN_6_O_6_ ([M+H]^+^) 825.0281, found 825.0319.

**Data for 6g:** White solid, yield: 87%, m.p. 232–235 °C; IR cm^−1^(KBr): 2923, 1776, 1603, 1480, 1441, 1009; ^1^H NMR (400 MHz CDCl_3_) *δ*: 7.87–7.90 (m, 4H, -Ph), 7.62–7.65 (m, 4H, -Ph), 7.30–7.32 (m, 2H, -Ph), 7.04 (d, *J* = 1.6 Hz, 1H, -Ph), 6.97 (d, *J* = 1.2 Hz, 1H, -Ph), 5.90–6.02 (m, 2H, -C*H*=CH_2_), 5.38 (s, 2H, -C*H*_2_-N), 5.33 (s, 2H, -C*H*_2_-N), 5.09–5.20 (m, 4H, -CH=C*H_2_*), 3.56 (d, *J* = 6.8 Hz, 2H, -C*H*_2_-CH=CH_2_), 3.43 (d, *J* = 6.4 Hz, 2H, -C*H*_2_-CH=CH_2_); ^13^C NMR (100 MHz CDCl_3_) *δ*: 165.46, 160.45, 153.93, 140.77, 140.30, 138.07, 137.49, 136.60, 134.43, 132.57, 132.53, 131.22, 130.63, 130.17, 128.58, 127.19, 124.60, 123.73, 123.63, 123.45, 122.00, 117.42, 116.88, 108.22, 106.89, 40.07, 36.87, 33.37; HRMS: calcd for C_38_H_27_^79^Br_2_N_6_O_6_ ([M+H]^+^) 821.0281, found 821.0351; calcd for C_38_H_27_^79^Br^81^BrN_6_O_6_ ([M+H]^+^) 823.0281, found 823.0339; calcd for C_38_H_27_^81^Br^81^BrN_6_O_6_ ([M+H]^+^) 825.0281, found 825.0326.

**Data for 6h:** White solid, yield: 49%, m.p. 160–163 °C; IR cm^−1^(KBr): 2967, 1770, 1619, 1470, 1442, 1340; ^1^H NMR (400 MHz CDCl_3_) *δ*: 7.98–8.04 (m, 2H, -Ph), 7.52–7.55 (m, 2H, -Ph), 7.28–7.32 (m, 4H, -Ph), 7.18–7.25 (m, 2H, -Ph), 7.05 (d, *J* = 1.2 Hz, 1H, -Ph), 6.98 (d, *J* = 1.2 Hz, 1H, -Ph), 5.91–6.00 (m, 2H, -C*H*=CH_2_), 5.40 (s, 2H, -C*H*_2_-N), 5.36 (s, 2H, -C*H*_2_-N), 5.09–5.20 (m, 4H, -CH=C*H_2_*), 3.56 (d, *J* = 6.8 Hz, 2H, -C*H*_2_-CH=CH_2_), 3.44 (d, *J* = 6.4 Hz, 2H, -C*H*_2_-CH=CH_2_); ^13^C NMR (100 MHz CDCl_3_) *δ*: 162.93, 162.84, 160.61, 160.53, 160.17 (d, *J_CF_* = 254.0 Hz), 153.93, 153.91, 140.84, 139.43, 138.60, 138.07, 137.41, 136.58, 134.41, 134.16, 134.09, 132.82, 131.20, 130.53, 130.15, 129.92, 128.19, 124.74, 123.60, 123.42, 119.95, 117.34, 117.19, 117.01, 116.83, 111.77, 108.30, 106.89, 40.08, 36.92, 36.84, 33.32; HRMS: calcd for C_38_H_27_F_2_N_6_O_6_ ([M+H]^+^) 701.1882, found 701.1959. 

**Data for 6i:** White solid, yield: 25%, m.p. 166–168 °C; IR cm^−1^(KBr): 2922, 1770, 1440, 870, 728, 681; ^1^H NMR (400 MHz CDCl_3_) *δ*: 7.80–7.83 (m, 2H, -Ph), 7.70–7.74 (m, 2H, -Ph), 7.45–7.49 (m, 2H, -Ph), 7.31–7.32 (m, 2H, -Ph), 7.20–7.25 (m, 2H, -Ph), 7.05 (d, *J* = 1.6 Hz, 1H, -Ph), 6.97 (d, *J* = 1.2 Hz, 1H, -Ph), 5.91–6.03 (m, 2H, -C*H*=CH_2_), 5.39 (s, 2H, -C*H*_2_-N), 5.34 (s, 2H, -C*H*_2_-N), 5.10–5.20 (m, 4H, -CH=C*H_2_*), 3.56 (d, *J* = 6.8 Hz, 2H, -C*H*_2_-CH=CH_2_), 3.44 (d, *J* = 6.8 Hz, 2H, -C*H*_2_-CH=CH_2_); ^13^C NMR (100 MHz CDCl_3_) *δ*: 165.19, 165.16, 162.81 (d, *J_CF_* = 252.0 Hz), 160.57, 160.53, 153.94, 153.89, 138.04, 137.48, 136.55, 134.37, 131.20, 131.09, 131.07, 131.01, 130.99, 130.49, 130.08, 124.98, 124.89, 124.62, 123.70, 123.63, 123.42, 122.99, 122.96, 119.54, 119.49, 119.33, 119.27, 117.40, 116.88, 114.36, 114.12, 108.20, 106.91, 40.08, 36.89, 36.82, 33.34; HRMS: calcd for C_38_H_27_F_2_N_6_O_6_ ([M+H]^+^) 701.1882, found 701.1959.

**Data for 6j:** White solid, yield: 50%, m.p. 168–170 °C; IR cm^−1^(KBr): 3074, 2970, 1778, 1611, 1499, 1442, 1237; ^1^H NMR (400 MHz CDCl_3_) *δ*: 8.01–8.05 (m, 4H, -Ph), 7.30–7.32 (m, 2H, -Ph), 7.15–7.20 (m, 4H, -Ph), 7.04 (s, 1H, -Ph), 6.97 (d, *J* = 1.2 Hz, 1H, -Ph), 5.90–6.02 (m, 2H, -C*H*=CH_2_), 5.37 (s, 2H, -C*H*_2_-N), 5.33 (s, 2H, -C*H*_2_-N), 5.10–5.20 (m, 4H, -CH=C*H_2_*), 3.56 (d, *J* = 6.8 Hz, 2H, -C*H*_2_-CH=CH_2_), 3.43 (d, *J* = 6.8 Hz, 2H, -C*H*_2_-CH=CH_2_); ^13^C NMR (100 MHz CDCl_3_) *δ*: 165.37, 165.34, 165.14 (d, *J_CF_* = 255.0 Hz), 160.23, 160.22, 153.94, 153.91, 140.80, 138.01, 137.42, 136.54, 134.36, 131.20, 130.50, 130.10, 129.62, 129.52, 129.51, 124.60, 123.67, 123.63, 123.41, 119.44, 119.41, 117.39, 116.84, 116.66, 116.65, 116.44, 116.43, 108.22, 106.90, 40.07, 36.88, 36.83, 33.33; HRMS: calcd for C_38_H_27_F_2_N_6_O_6_ ([M+H]^+^) 701.1882, found 701.1951.

**Data for 6k:** White solid, yield: 64%, m.p. 181–184 °C; IR cm^−1^(KBr): 2923, 1780, 1440, 1341, 1064, 730; ^1^H NMR (400 MHz CDCl_3_) *δ*: 7.87 (d, *J* = 7.6 Hz, 2H, -Ph), 7.37–7.43 (m, 2H, -Ph), 7.28–7.33 (m, 6H, -Ph), 7.04 (d, *J* = 1.2 Hz, 1H, -Ph), 6.98 (d, *J* = 1.2 Hz, 1H, -Ph), 5.90–6.00 (m, 2H, -C*H*=CH_2_), 5.39 (s, 2H, -C*H*_2_-N), 5.34 (s, 2H, -C*H*_2_-N), 5.09–5.20 (m, 4H, -CH=C*H_2_*), 3.56 (d, *J* = 6.8 Hz, 2H, -C*H*_2_-CH=CH_2_), 3.43 (d, *J* = 6.8 Hz, 2H, -C*H*_2_-CH=CH_2_), 2.67 (s, 3H, -C*H_3_* ), 2.62 (s, 3H, -C*H*_3_); ^13^C NMR (100 MHz CDCl_3_) *δ*: 166.33, 166.28, 159.74, 159.71, 153.98, 153.94, 140.80, 138.72, 138.03, 137.35, 136.56, 134.39, 131.84, 131.81, 131.73, 131.69, 131.20, 130.56, 130.16, 129.18, 126.25, 124.59, 123.62, 123.60, 123.38, 122.16, 117.34, 116.82, 108.20, 106.88, 40.07, 36.90, 36.85, 33.33, 22.09, 22.06; HRMS: calcd for C_40_H_33_N_6_O_6_ ([M+H]^+^) 693.2383, found 693.2457.

**Data for 6l:** White solid, yield: 20%, m.p. 150–153 °C; IR cm^−1^(KBr): 2924, 1781, 1441, 1340, 1064, 724; ^1^H NMR (400 MHz CDCl_3_) *δ*: 7.79–7.85 (m, 4H, -Ph), 7.31–7.37 (m, 6H, -Ph), 7.03 (d, *J* = 1.6 Hz, 1H, -Ph), 6.97 (d, *J* = 1.2 Hz, 1H, -Ph), 5.90–6.02 (m, 2H, -C*H*=CH_2_), 5.38 (s, 2H, -C*H*_2_-N), 5.33 (s, 2H, -C*H*_2_-N), 5.09–5.20 (m, 4H, -CH=C*H_2_*), 3.56 (d, *J* = 6.4 Hz, 2H, -C*H*_2_-CH=CH_2_), 3.43 (d, *J* = 6.8 Hz, 2H, -C*H*_2_-CH=CH_2_), 2.41 (s, 3H, -C*H_3_*), 2.38 (s, 3H, -C*H*_3_); ^13^C NMR (100 MHz CDCl_3_) *δ*: 166.35, 166.29, 160.11, 160.08, 153.98, 153.95, 140.78, 139.08, 139.04, 138.01, 137.38, 136.54, 134.39, 133.11, 133.06, 131.22, 130.51, 130.12, 129.01, 127.63, 124.63, 124.33, 123.60, 123.39, 122.94, 117.35, 116.83, 108.23, 106.89, 40.06, 36.93, 36.87, 33.33, 21.29, 21.27; HRMS: calcd for C_40_H_33_N_6_O_6_ ([M+H]^+^) 693.2383, found 693.2445.

**Data for 6m:** White solid, yield: 32%, m.p. 212–215 °C; IR cm^−1^(KBr): 2922, 1776, 1615, 1499, 1443, 1343; ^1^H NMR (400 MHz CDCl_3_) *δ*: 7.88–7.92 (m, 4H, -Ph), 7.28–7.31 (m, 6H, -Ph), 7.03 (s, 1H, -Ph), 6.97 (d, *J* = 0.8 Hz, 1H, -Ph), 5.90–6.02 (m, 2H, -C*H*=CH_2_), 5.37 (s, 2H, -C*H*_2_-N), 5.32 (s, 2H, -C*H*_2_-N), 5.09–5.20 (m, 4H, -CH=C*H_2_*), 3.56 (d, *J* = 6.4 Hz, 2H, -C*H*_2_-CH=CH_2_), 3.43 (d, *J* = 6.8 Hz, 2H, -C*H*_2_-CH=CH_2_), 2.41 (s, 3H, -C*H_3_*), 2.40 (s, 3H, -C*H_3_*); HRMS: calcd for C_40_H_33_N_6_O_6_ ([M+H]^+^) 693.2383, found 693.2455.

**Data for 6n:** White solid, yield: 64%, m.p. 95–98 °C; IR cm^−1^(KBr): 2926, 1783, 1607, 1445, 1261, 1014, 749; ^1^H NMR (400 MHz CDCl_3_) *δ*: 7.86–7.89 (m, 2H, -Ph), 7.45–7.52 (m, 2H, -Ph), 7.28-7.31 (m, 2H, -Ph), 6.96-7.04 (m, 6H, -Ph), 5.89-6.02 (m, 2H, -C*H*=CH_2_), 5.38 (s, 2H, -C*H*_2_-N), 5.34 (s, 2H, -C*H*_2_-N), 5.08–5.19 (m, 4H, -CH=C*H_2_*), 3.91 (s, 3H, -OC*H_3_*), 3.83 (s, 3H, -OC*H*_3_), 3.56 (d, *J* = 6.4 Hz, 2H, -C*H*_2_-CH=CH_2_), 3.42 (d, *J* = 6.8 Hz, 2H, -C*H*_2_-CH=CH_2_); ^13^C NMR (100 MHz CDCl_3_) *δ*: 164.85, 159.88, 158.02, 157.96, 153.98, 140.78, 138.02, 137.29, 136.57, 134.44, 133.59, 133.54, 131.20, 130.63, 130.60, 130.24, 124.57, 123.58, 123.46, 123.32, 120.77, 117.30, 116.78, 112.23, 111.97, 111.91, 108.32, 106.95, 55.98, 55.87, 40.06, 36.99, 36.94, 33.33; HRMS: calcd for C_40_H_33_N_6_O_8_ ([M+H]^+^) 725.2282, found 725.2355.

**Data for 6o:** White solid, yield: 83%, m.p. 100–103 °C; IR cm^−1^(KBr): 2963, 1782, 1465, 1143, 1045; ^1^H NMR (400 MHz CDCl_3_) *δ*: 7.52–7.58 (m, 4H, -Ph), 7.35–7.41 (m, 2H, -Ph), 7.31 (d, *J* = 0.8 Hz, 2H, -Ph), 7.03–7.07 (m, 3H, -Ph), 6.97 (d, *J* = 1.2 Hz, 1H, -Ph), 5.94–6.00 (m, 2H, -C*H*=CH_2_), 5.38 (s, 2H, -C*H*_2_-N), 5.33 (s, 2H, -C*H*_2_-N), 5.09–5.20 (m, 4H, -CH=C*H_2_*), 3.86 (s, 3H, -OC*H_3_*), 3.83 (s, 3H, -OC*H*_3_), 3.56 (d, *J* = 6.8 Hz, 2H, -C*H*_2_-CH=CH_2_), 3.43 (d, *J* = 6.4 Hz, 2H, -C*H*_2_-CH=CH_2_); ^13^C NMR (100 MHz CDCl_3_) δ: 166.13, 166.08, 160.23, 160.20, 159.99, 159.95, 153.95, 140.79, 138.01, 137.38, 136.54, 134.38, 131.21, 130.52, 130.29, 130.13, 124.63, 124.16, 123.61, 123.39, 119.58, 118.84, 118.80, 117.36, 116.84, 111.70, 111.61, 108.22, 106.89, 55.53, 40.06, 36.94, 36.88, 33.33; HRMS: calcd for C_40_H_33_N_6_O_8_ ([M+H]^+^) 725.2282, found 725.2354. 

**Data for 6p:** White solid, yield: 50%, m.p. 228–230 °C; IR cm^−1^(KBr): 2928, 1774, 1612, 1498, 1262, 1175; ^1^H NMR (400 MHz CDCl_3_) *δ*: 7.93–7.97 (m, 4H, -Ph), 7.30 (s, 2H, -Ph), 7.03(s, 1H, -Ph), 6.95–6.99 (m, 5H, -Ph), 5.90–6.00 (m, 2H, -C*H*=CH_2_), 5.36 (s, 2H, -C*H*_2_-N), 5.31 (s, 2H, -C*H*_2_-N), 5.09–5.20 (m, 4H, -CH=C*H_2_*), 3.86 (s, 3H, -OC*H_3_*), 3.85 (s, 3H, -OC*H*_3_), 3.56 (d, *J* = 6.4 Hz, 2H, -C*H*_2_-CH=CH_2_), 3.43 (d, *J* = 6.4 Hz, 2H, -C*H*_2_-CH=CH_2_); ^13^C NMR (100 MHz CDCl_3_) *δ*: 166.12, 166.07, 162.74, 162.71, 159.66, 159.63, 153.95, 140.77, 138.01, 137.35, 136.57, 134.40, 131.22, 130.54, 130.15, 128.99, 124.59, 123.54, 123.36, 117.33, 116.80, 115.54, 114.55, 108.26, 106.91, 55.48, 40.06, 36.87, 33.33; HRMS: calcd for C_40_H_33_N_6_O_8_ ([M+H]^+^) 725.2282, found 725.2358. 

### 3.4. Cell Culture

HEK-293T cells were purchased from the American Type Culture Collection (ATCC, Rockville, MD, USA). HEK-293T-ACE2^h^ cells were constructed by Sino Biological (Beijing, China). All the cell lines were cultured in Dulbecco Modified Eagle Medium (DMEM) supplemented with 10% fetal bovine serum, the antibiotics penicillin (50 U/mL) and streptomycin (50 μg/mL, Invitrogen, Paisley, Scotland, UK) at 37 °C in a humidified atmosphere of 5% CO_2_. Besides, 100 μg/mL hygromycin was used for the culture of HEK-293T-ACE2^h^ cells.

### 3.5. Cytotoxicity Assay

The cytotoxic effect of honokiol derivatives on HEK-293T-ACE2^h^ cells was evaluated by MTT assay. HEK-293T-ACE2^h^ cells were seed into 96-well plate at a density of 1×10^4^ cells per well and then treated with various concentrations of compounds for either 4 or 48 h. Then 10 μL of 3-(4, 5-dimethylthiazol-2-yl)-2, 5-diphenyltetrazolium bromide (MTT) (5 mg/mL) solution was added to each well for another 4 h incubation. The supernatants were then removed, and the formed formazan crystals were dissolved in 100 μL DMSO and the plate was agitated for 10 min. Relative cell viability was assessed by measuring the absorbance at 570 nm by using a microplate reader (Infinite M200 PRO Microplate Reader, Tecan, Swiss). The survival rate of HEK-293T-ACE2^h^ cells was calculated using the following formula: [(OD_treated_-OD_blank_) / (OD_control_-OD_blank_)] × 100%. The 50% toxic concentration (TC_50_) for 48 h was calculated by the GraphPad Prism 8.0 software (La Jolla, CA, USA). The maximum nontoxic concentration (CC_0_) for 4 h was determined as the maximum tested concentration in which the cell viability was 100%. Data were obtained from three independent experiments.

### 3.6. Production of SARS-CoV-2 Spike Pseudoviruses

HIV-1 reporter pseudoviruses expressing SARS-CoV-2 spike protein and luciferase were constructed by using pNL4-3.Luc.R-E- plasmid and pcDNA3.1-SARS-CoV-2-Spike-Myc plasmid [14,15,16]. The plasmid of pNL4-3.Luc.R-E- was bought from the NIH AIDS repository. The plasmid of pcDNA3.1-SARS-CoV-2-Spike-Myc was purchased from Beyotime (Shanghai, China). HEK-293T cells were seeded into six-well plates at a density of 5 × 10^5^ cells per well. When the cells’ confluency reached 60–80% after 24 h incubation, two plasmids of pNL4-3.Luc.R-E- and pcDNA3.1-SARS-CoV-2-Spike-Myc were co-transfected into the cells with 125 μL of Opti-MEM and 6 μL of Lipo8000 reagent (Beyotime, China). After 24 h post-transfection, supernatants were harvested and then centrifuged at 1500 rpm/min to remove cell debris. The infectious competency of 30 µL of produced pseudovirus to host cells was measured with reference to that of SARS-CoV-2 spike pseudovirus (10^10^ copies/ mL, PSV001) from Sino Biological as follows. A series of 0, 5, 10, 20 and 50 µL of SARS-CoV-2 spike pseudovirus (10^10^ copies/ mL) from Sino Biological was diluted by culture medium to a final volume of 100 µL, and subsequently utilized to infect the HEK-293T-ACE2^h^ cells for 2 h at 37 °C in duplicates. After that, the cells were replaced with the fresh culture medium to remove the inoculum and were further incubated for 48 h. Subsequently, 100 µL of cell lysate buffer was added into each well, and the plate was gently agitated for 10 min. The luciferase luminescence was measured at 578 nm by a microplate reader (SpectraMax iD5 Multi-Mode Microplate Reader, Molecular Devices, San Jose, CA, USA) immediately after 100 µL of luminescence solution was added into each well according to the instruction from the Firefly Luciferase Reporter Gene Assay Kit (Beyotime, Shanghai, China). A standard curve of luciferase luminescence in response to pseudovirus copies was generated as the viral infection equation, Y = 197.17 x – 62.821, with regression coefficient R^2^ value of 0.9996 [28]. Simultaneously, the 30 µL supernatant of produced pseudovirus was used to infect the host cells following the above experimental procedure in hexaplicates. From the established standard curve, the infectious competency of 30 uL supernatant solution of produced pseudovirus was determined as equivalent to that of 9.12 ± 0.88 × 10^7^ copies (n = 6) of SARS-CoV-2 pseudovirus from Sino Biological. Thus, the pseudovirus utilized in our experiments was considered to possess equivalent infectious competency to 9.12 ± 0.88 × 10^7^ copies of SARS-CoV-2 pseudovirus from Sino Biological for HEK-293T-ACE2^h^ cells under tested conditions. The quantified pseudovirus made in our lab was thus utilized for the subsequent infection experiments to HEK-293T-ACE2^h^ cells.

### 3.7. The Detection of SARS-CoV-2 Spike Pseudovirus Entry into HEK-293T-ACE2^h^ Cells

The tested compounds were used at concentrations ranging from 1.56 to 100 µM at two-fold serial dilutions. An antibody of SARS-CoV-2 (Sino Biological, Beijing, China) was employed as a positive viral entry inhibitor with tested concentrations ranging from 1.875 to 60 nM. HEK-293T-ACE2^h^ cells (1 × 10^4^ in 100 uL DMEM) were seeded in 96-well plates, and cultured in a 37 °C incubator containing 5% CO_2_ for 24 h. Then, the culture medium was replaced by 100 uL compounds-containing DMEM medium for 2 h incubation in the 37 °C incubator. After 2 h incubation, 30 μL pseudovirus (MOI = 100 virus particles/cell), 20 μL media, and 50 μL two-fold concentration of compounds were simultaneously added into each well for another 2 h incubation at 37 °C. After removal of the inoculum, cells were overlaid with 100 μL fresh DMEM, and incubated continuously at 37 °C for 48 h. The culture medium was aspirated and the cells were washed once by PBS. Then, 100 μL of cell lysate buffer was added into each well, and the plate was agitated for 10 min. The cell lysate was completely transferred to an opaque 96-well white solid plate. The luciferase luminescence was measured at 578 nm by a microplate reader (SpectraMax iD5 Multi-Mode Microplate Reader, Molecular Devices, San Jose, CA, USA) immediately after 100 μL of luminescence solution was added into each well according to the instruction from the Firefly Luciferase Reporter Gene Assay Kit (Beyotime, Shanghai, China). HEK-293T-ACE2^h^ cells infected only with SARS-CoV-2 spike pseudovirus were considered as control group, and the luciferase luminescence value of the control was defined as 100%. The values of luminescence of compounds-treated groups were normalized accordingly [29]. A standard curve was simultaneously carried out by using SARS-CoV-2 pseudovirus (1 × 10^10^ copies/mL from Sino Biological) to infect HEK-293T-ACE2^h^ cells in each independent experiment. The inhibitory activity of tested compounds against pseudovirus entry was expressed as IC_50_ value which was defined as the concentration that inhibits 50% of the pseudovirus from entering the target cell. The IC_50_ was calculated by using the GraphPad Prism 8.0 software (La Jolla, CA, USA). All the data were expressed as means ± standard deviation (SD) from three independent experiments.

### 3.8. Biolayer Interferometry (BLI) Binding Assay

The binding kinetics of compounds with the protein of recombinant histidine-tagged human ACE2 (Sino Biological, Beijing, China) were analyzed by bio-layer interferometry on an Octet system (Octet RED96, ForteBio, Fremont, CA, USA) [22,23]. Histidine-tagged human ACE2 protein (25 μg/mL aqueous solution) was immobilized on a nickel charged nitrilotriacetic acid (Ni-NTA) biosensor (Fortebi, Fremont, CA, USA) by a protein-loading program of the instrument. Each stock solution of compounds (10 mM in DMSO) was serially diluted by PBS buffer with a final DMSO concentration of 1.0 %. The SARS-CoV-2 spike RBD protein (Sino Biological, Beijing, China) was utilized as a positive binding protein with tested concentrations ranging from 0.03125 to 1 nM. Both protein-immobilized and empty biosensors were equilibrated in PBS buffer for 10 min at room temperature before preceding data acquisition, and all experiments were performed at 30 °C. Both the protein-coated and empty biosensors were dipped in wells containing serially diluted samples. The signal of background was subtracted from all samples by dipping a protein-immobilized biosensor in the blank buffer. Under the same conditions, empty biosensors were also dipped in serially diluted samples for reference subtraction. The subtracted sensorgrams were then fitted to a 1:1 binding model by using Octet Data Analysis Software v11.1 (ForteBio, Torrance, CA, USA) to calculate the resulting equilibrium dissociation constant (K_D_) values for the interaction. The interaction of **6p** with a recombinant histidine-tagged SARS-CoV-2 spike RBD protein (25 μg/mL aqueous solution, Sino Biological, Beijing, China) was also analyzed with the same experimental procedure by BLI binding assay.

### 3.9. Docking Studies

CDOCKER of Discovery Studio software was employed to perform molecular docking analysis on the structure of protein of human ACE2 bound with its inhibitor (PDB ID: 1R4L). A docking pocket was generated based on the position of the inhibitor of human ACE2 [27]. The binding modes were analyzed with the Discovery Studio software.

### 3.10. ELISA Assay

An ELISA assay that mimics the viral spike RBD-ACE2 binding process was employed to investigate the compounds’ interventive effect on the binding of spike RBD-ACE2 by a SARS-CoV-2 (2019-nCoV) inhibitor screening ELISA kit (Sino Biological, Beijing, China) with a slight optimization of the kit’s experimental protocol. Briefly, the wells pre-coated with SARS-CoV-2 spike RBD-mFc recombinant protein were washed three times with washing buffer (300 µL/ well). At the same time, human ACE2 protein with His Tag (100 µL) was incubated with equal volume of compounds for 1 h. The final concentration of tested compounds was 12.5, 25, and 50 µM in the mixture solution, respectively. The mixture of compounds-incubated ACE2 solution (200 µL) was added into each well for another 1 h incubation. Then, the solution in wells was disposed and the wells were washed three times. Anti-His Tag antibody (HRP, 100 µL) was added into each well for 1 h incubation at room temperature. Subsequently, each well was washed three times again, and substrate solution (200 µL) was added into each well for 15 minutes incubation with protection from light. Finally, the optical density of each well was measured at 450 nm by a microplate reader after the stop solution (50 µL) was supplemented to each well. The average OD value of wells with only human ACE2 protein was defined as the control group with the assumption that the pre-coated spike RBD was completely occupied by human ACE2 protein. The inhibitory rate (IR) of compound was calculated as follows: IR (%) = (OD_control_ − OD_compound-treated_) / OD_control_ × 100. The SARS-CoV-2 antibody inhibitor was provided by the ELISA kit, and its IC_50_ value was measured to be 2.59 nM in this assay. All the data were from three independent experiments.

### 3.11. Statistical Analysis

Statistical comparisons were calculated by Student’s t-test analysis. *p* < 0.05 was regarded as statistically significant.

## 4. Conclusions

In summary, sixteen derivatives of honokiol have been successfully synthesized. Compounds **6p** showed the most potent antiviral entry activity as a human ACE2 blocker. Moreover, **6a** and **6p** had higher biological safety for the host cells than the parental honokiol. This was the first report about the antiviral entry activity of modified honokiol derivative **6p** against SARS-CoV-2 pseudovirus via targeting human ACE2 receptor. The inhibitory effect of **6p** on SARS-CoV-2 viral strains needs further study in a biosafety level 3 facility. This research may contribute to the discovery of potential viral entrance inhibitors for SARS-CoV-2 virus.

## Figures and Tables

**Table 1:** Antiviral properties of honokiol derivatives **6a-p** against the entry of SARS-CoV-2 spike pseudovirus into HEK-293T-ACE2^h^ cells.

Compounds	CC_0_ ^1^ (µM)	TC_50_ ^2^ (µM)	IC_50_^3^ (µM)	SI ^4^
**6a**	>100	>100	29.23 ± 0.71	>3.42
**6b**	>100	>100	>100	ND
**6c**	>100	>100	>100	ND
**6d**	50	ND	>50	ND
**6e**	>100	>100	>100	ND
**6f**	50	ND	>50	ND
**6g**	50	ND	>50	ND
**6h**	>100	>100	61.58 ± 1.21	>1.62
**6i**	>100	>100	>100	ND
**6j**	50	ND	>50	ND
**6k**	>100	>100	>100	ND
**6l**	>100	>100	>100	ND
**6m**	50	ND	>50	ND
**6n**	50	ND	>50	ND
**6o**	>100	>100	>100	ND
**6p**	>100	>100	9.82 ± 1.16	>10.18
honokiol	50	48.23 ± 1.19	>50	ND
Antibody inhibitor ^5^	0.10	ND	0.035 ± 0.001	ND

^1^ CC_0_ means the maximum nontoxic concentration of compounds for 4 h incubation with cells; ^2^ TC_50_ is the necessary concentration that caused 50% host cells’ death for 48 h treatment; ^3^ IC_50_ stands for the compounds’ concentration that inhibited 50% viral entry into host cells. ^4^ SI means selectivity index that measures compounds’ window between cytotoxicity and antiviral activity by dividing the TC_50_ value into the IC_50_ value (TC_50_/ IC_50_); ^5^ SARS-CoV-2 antibody was used as a positive viral entry inhibitor; ND means not determined. All the data are expressed as means ± standard deviation (SD) from three independent experiments.

**Table 2:** Equilibrium dissociation constant (K_D_) values of **6a**, **6p,** and honokiol with human ACE2 protein by BLI binding assay and their binding energy by a molecular docking study.

Compounds	K_D_ Values (µM)	Binding Energy (kcal/mol)
SARS-CoV-2 spike RBD	0.01182 ± 0.00107	—
**6a**	8.41 ± 0.43	−59.849
**6p**	6.87 ± 0.29	−65.339
honokiol	16.98 ± 3.40	−31.963

## Data Availability

The data presented in this study are available on request from the corresponding author.

## Supplementary Materials

The following are available online at https://www.mdpi.com/article/10.3390/ph14090885/s1. Figures S1–S47: HRMS, ^1^H, and ^13^C NMR spectra for compounds **6a**–**p**.
