# Supplementary material for: Synthesis and Biological Evaluation of Honokiol Derivatives Bearing 3-((5-phenyl-1,3,4-oxadiazol-2-yl)methyl)oxazol-2(3H)-ones as Potential Viral Entry Inhibitors against SARS-CoV-2
Source: Pharmaceuticals (Basel). 2021 Aug 31;14(9):885. doi: 10.3390/ph14090885 (PMC8471451; doi:10.3390/ph14090885)
Supplement: Supplementary file 1 [file pharmaceuticals-14-00885-s001.zip › pharmaceuticals-1355967-supplementary.pdf]

Supplementary data

Synthesis and biological evaluation of honokiol derivatives bearing 3-((5-phenyl-1,3,4-oxadiazol-2-yl)methyl)oxazol-2(3*H*)-ones as potential viral entry inhibitors against SARS-CoV-2

Yong Guo [1, 2, #], Jieru Meng [1, #], Jiazheng, Liu[1], Ting Xu[1], Zhiyuang Zheng[1], Zhi-Hong Jiang[1] and Li-Ping Bai [1, *]

[1] State key laboratory of of Quality Research in Chinese Medicine, Macau Institute for Applied Research in Medicine and Health, Guangdong-Hong Kong-Macao Joint Laboratory of Respiratory Infectious Disease, Macau University of Science and Technology, Taipa, Macau, 999078, China; yguo@must.edu.mo; 1909853gct20002@student.must.edu.mo; 2009853DCT20001@student.must.edu.mo; 1909853ect30003@student.must.edu.mo; 1709853ect30001@student.must.edu.mo; zhjiang@must.edu.mo; lpbai@must.edu.mo

[2] School of Pharmaceutical Sciences, Zhengzhou University, Zhengzhou, 450001, Henan Province, China; yguo@must.edu.mo

* Correspondence: lpbai@must.edu.mo; Tel.: +853-8897-2403

[#] These authors contributed equally.

HRMS, ^{1}H NMR and ^{13}C NMR spectra for compounds 6a-p:

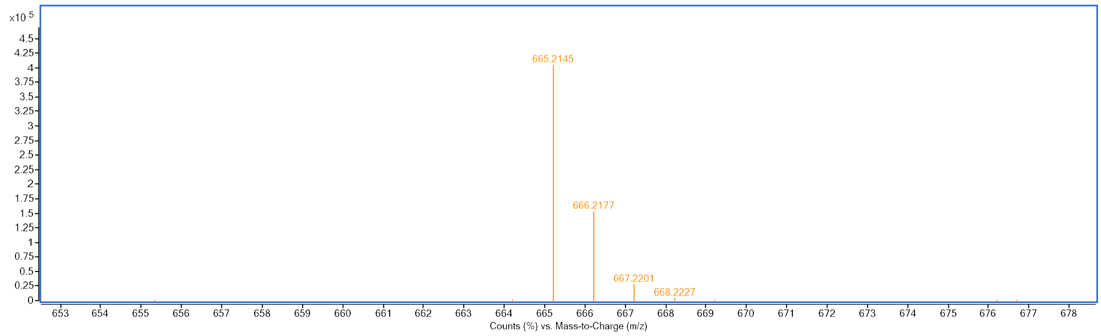

Figure S1. HRMS spectrum of **6a**

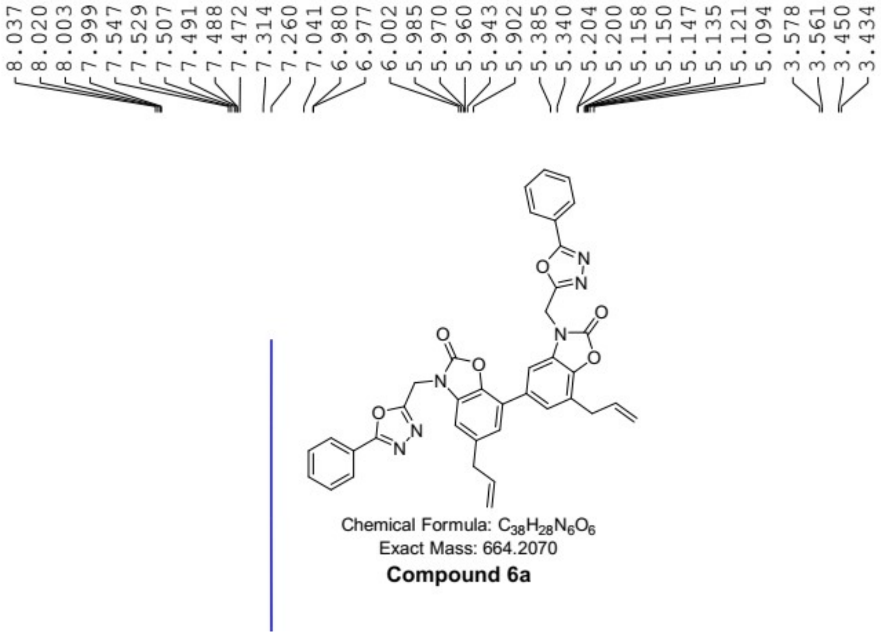

Figure S2. ^{1}H NMR spectrum of **6a**

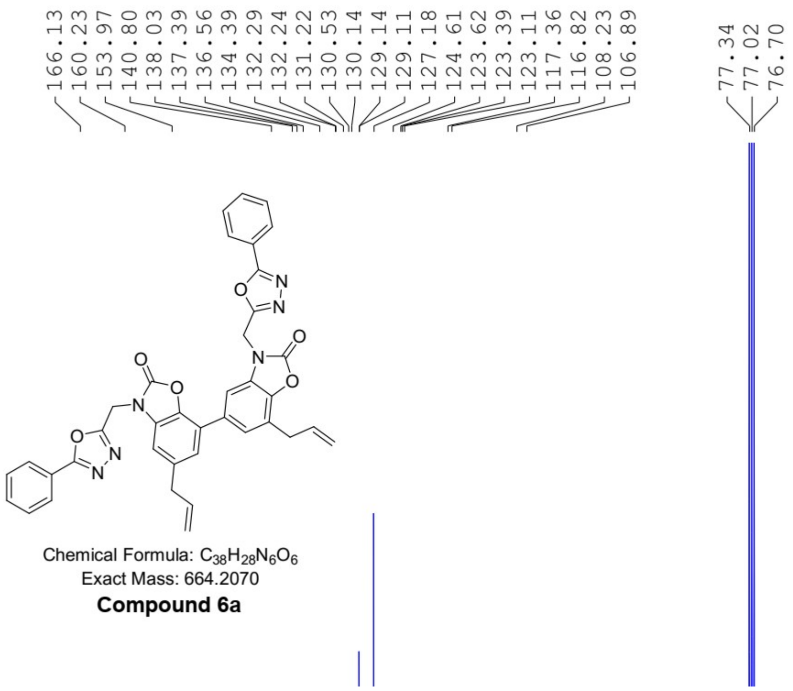

Figure S3. ^{13}C NMR spectrum of **6a**

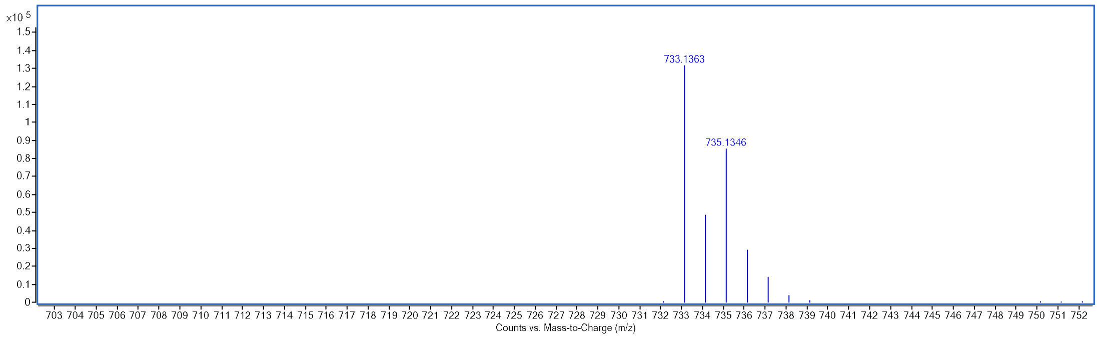

Figure S4. HRMS spectrum of **6b**

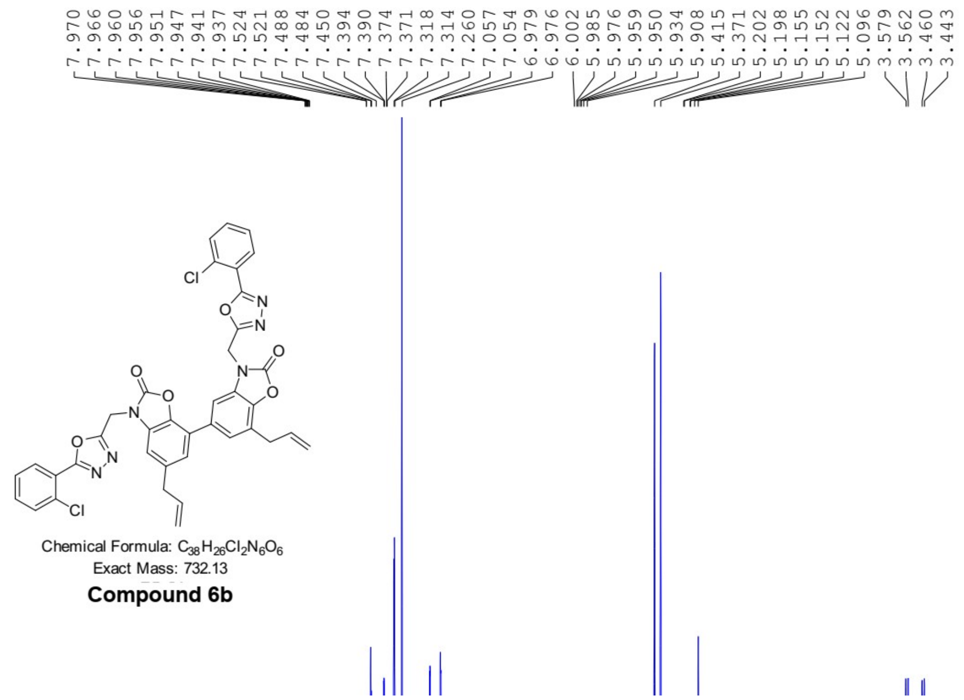

Figure S5. ^{1}H NMR spectrum of **6b**

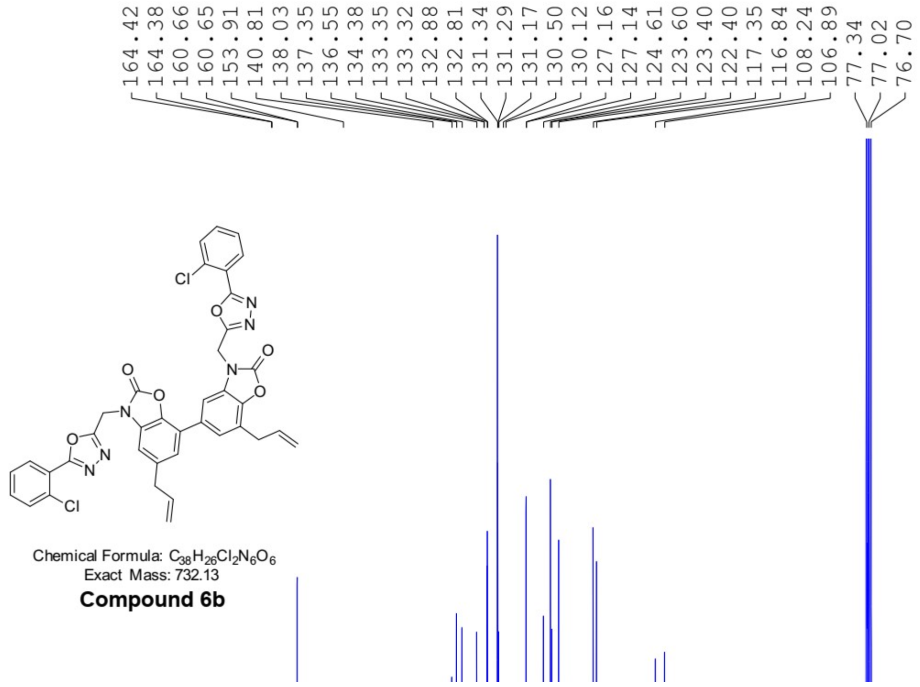

Figure S6. ^{13}C NMR spectrum of **6b**

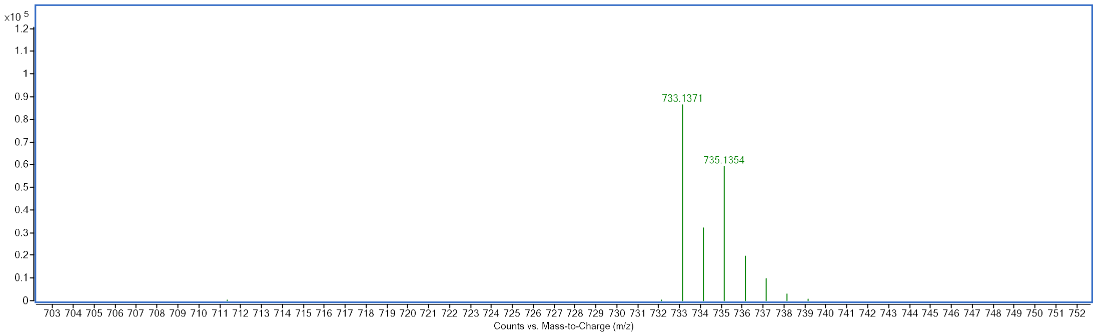

Figure S7. HRMS spectrum of **6c**

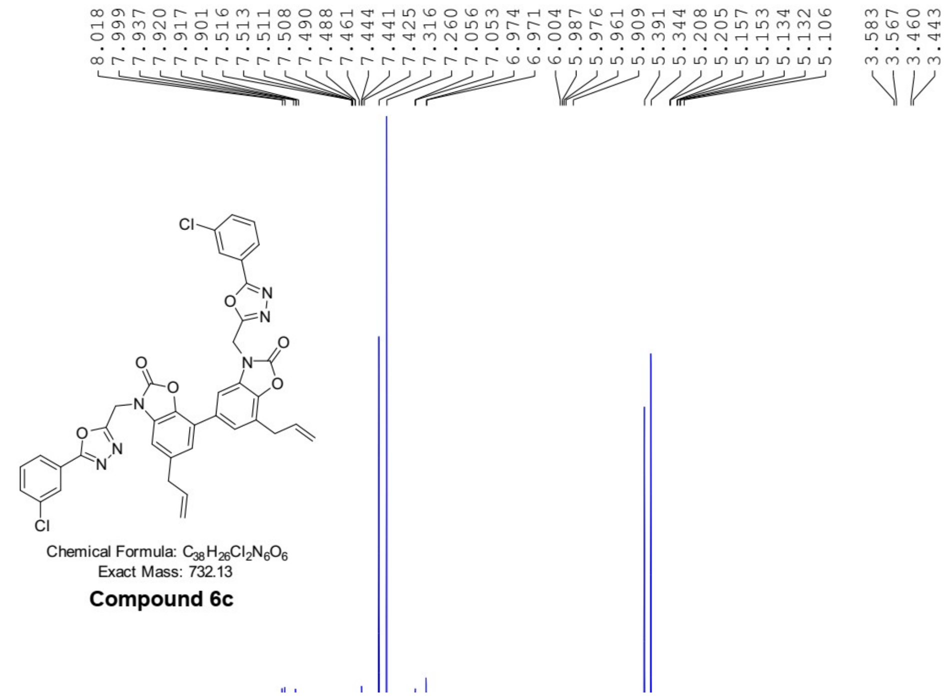

Figure S8. ^{1}H NMR spectrum of **6c**

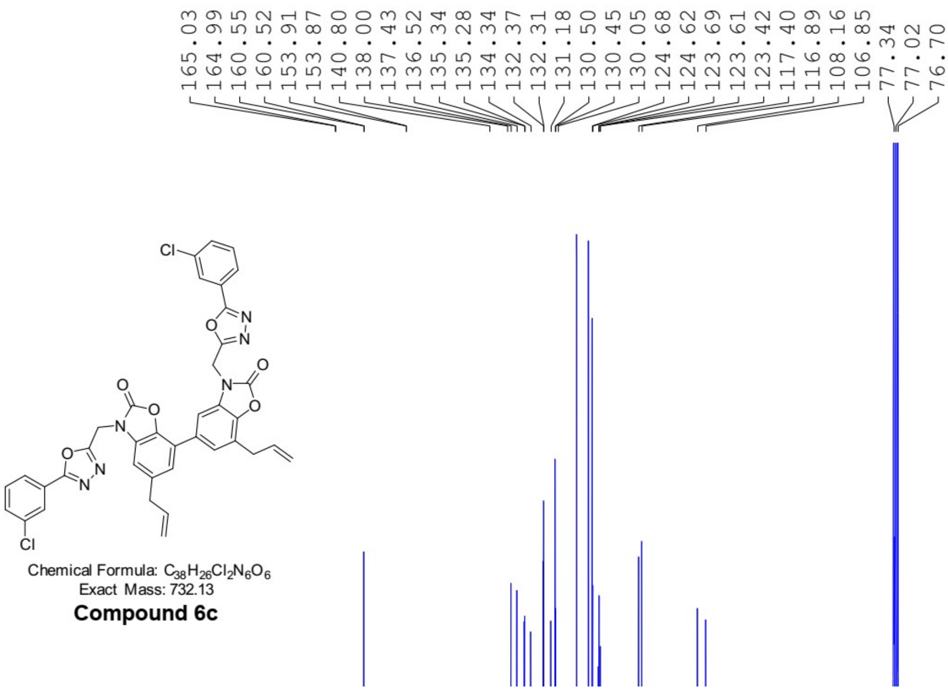

Figure S9. ^{13}C NMR spectrum of **6c**

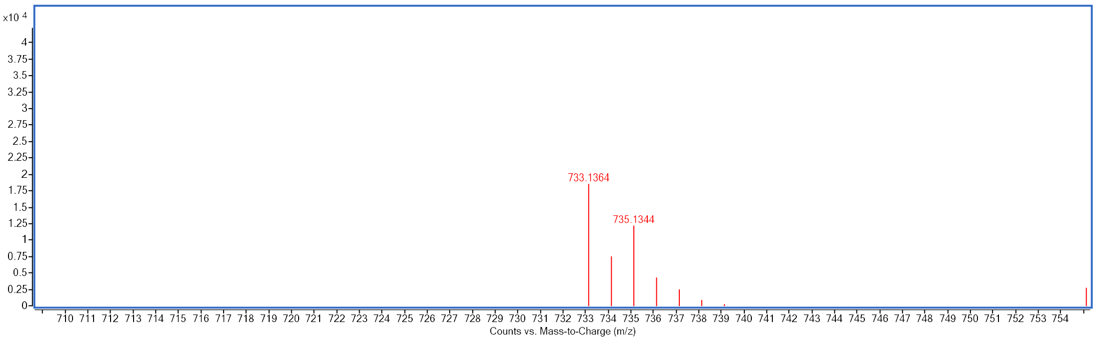

Figure S10. HRMS spectrum of **6d**

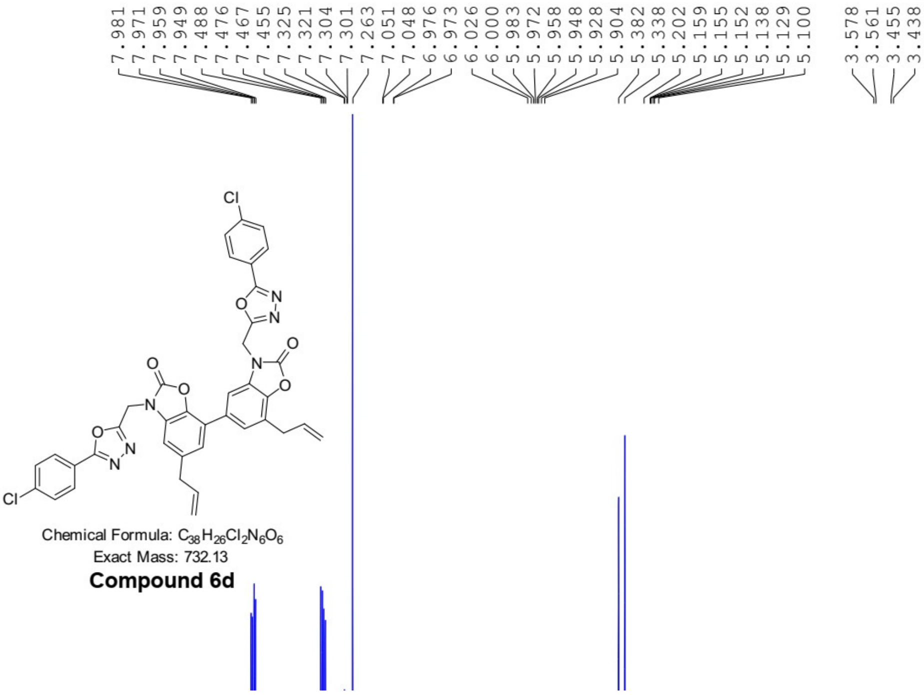

Figure S11. ^{1}H NMR spectrum of **6d**

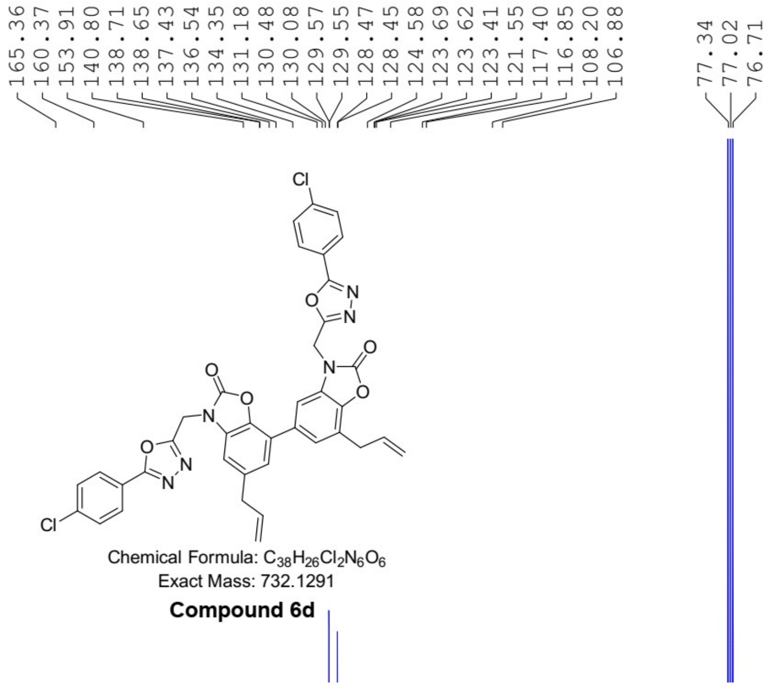

Figure S12. ^{13}C NMR spectrum of **6d**

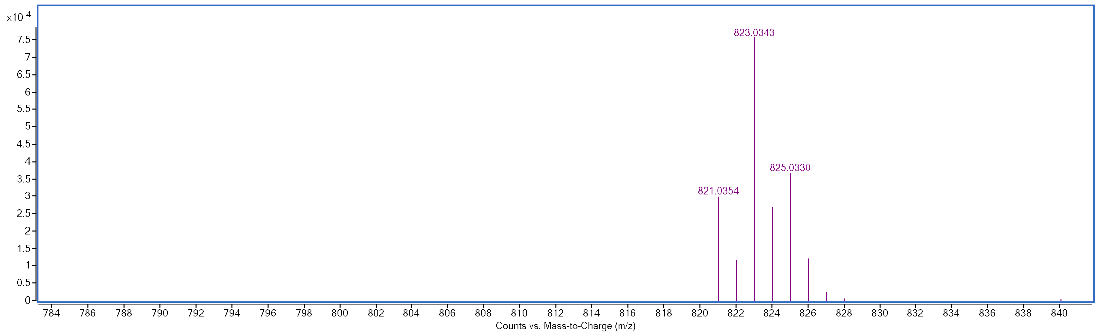

Figure S13. HRMS spectrum of **6e**

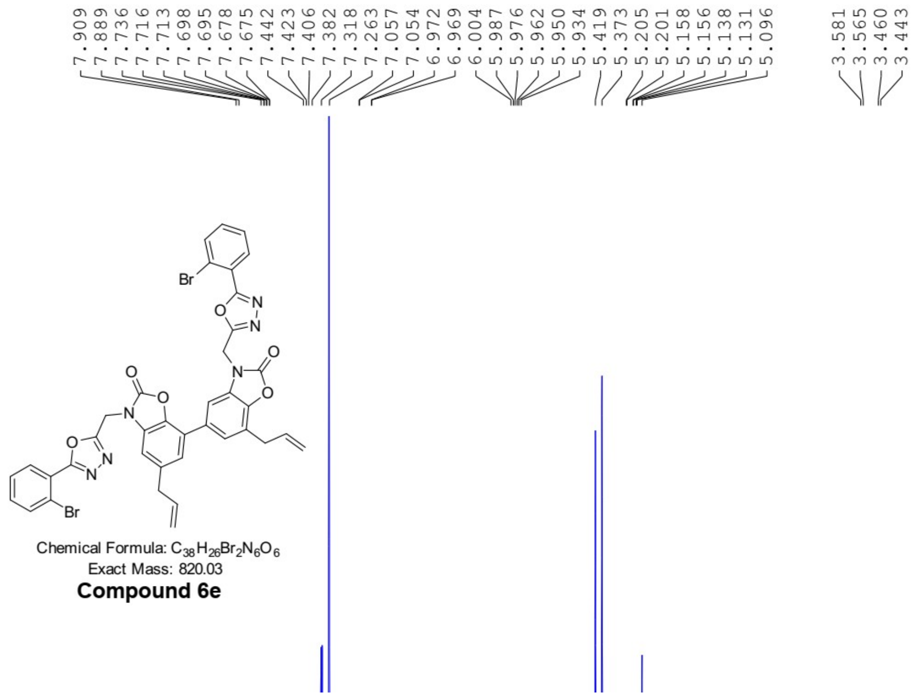

Figure S14. ^{1}H NMR spectrum of **6e**

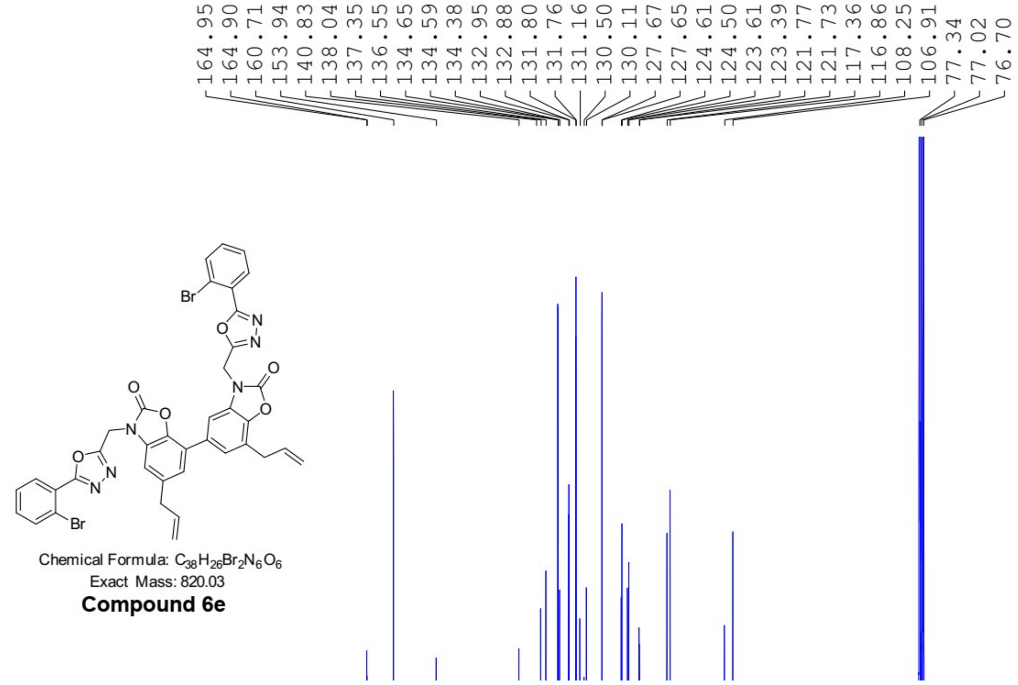

Figure S15. ^{13}C NMR spectrum of **6e**

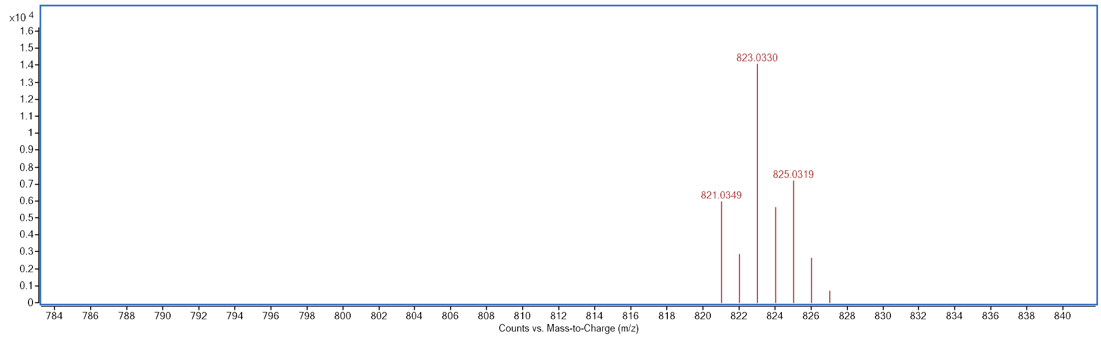

Figure S16. HRMS spectrum of **6f**

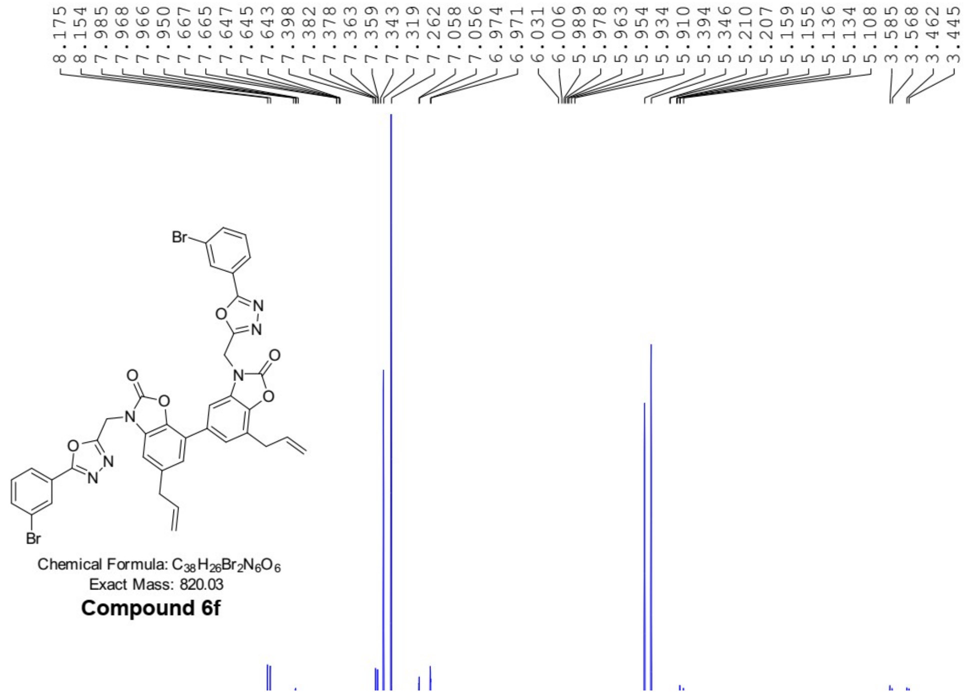

Figure S17. ^{1}H NMR spectrum of **6f**

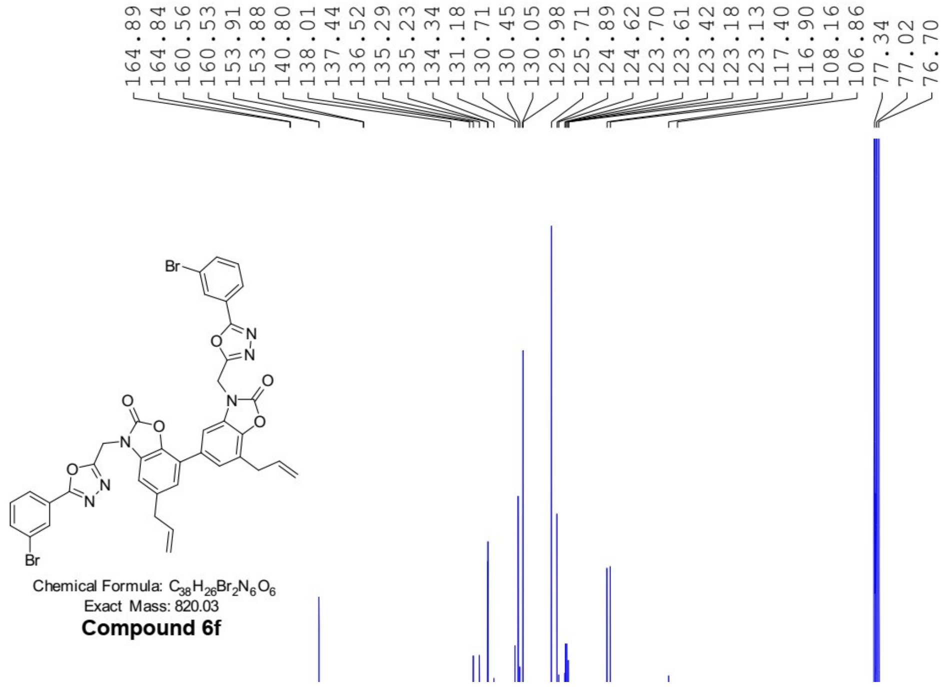

Figure S18. ^{13}C NMR spectrum of **6f**

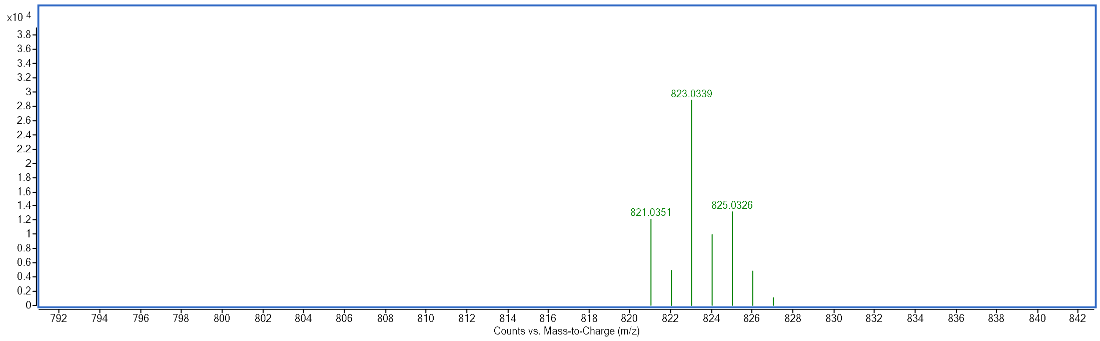

Figure S19. HRMS spectrum of **6g**

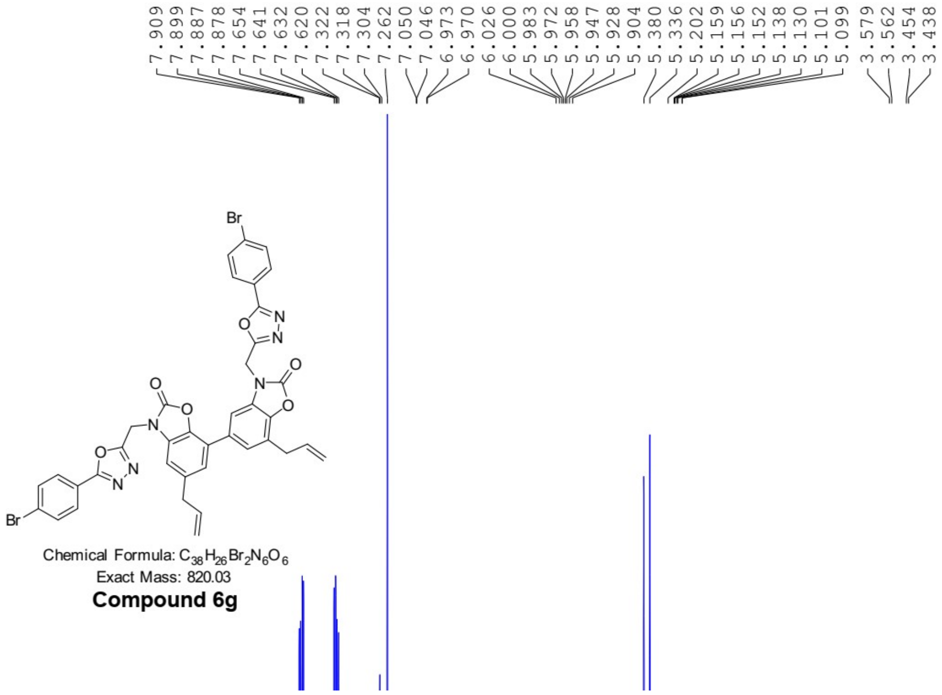

Figure S20. ^{1}H NMR spectrum of **6g**

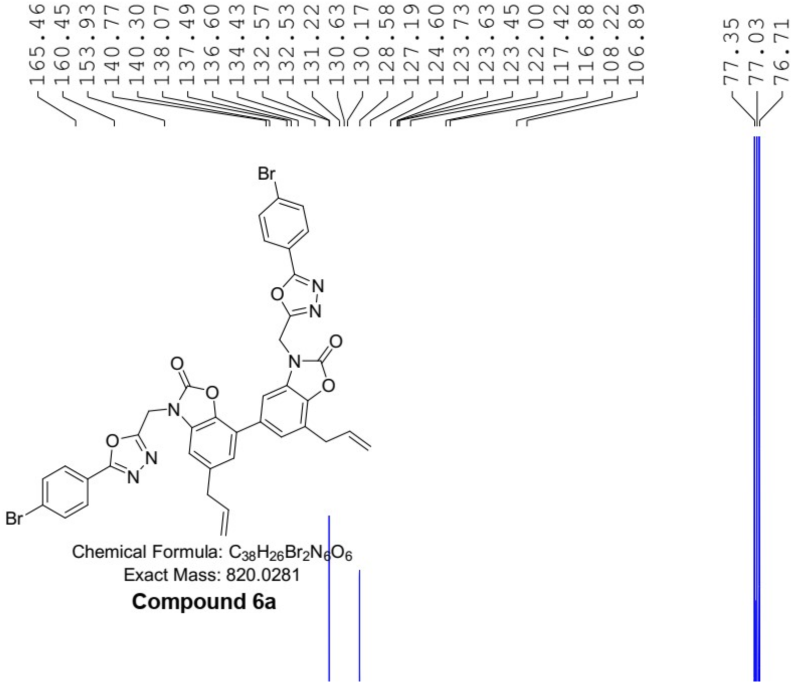

Figure S21. ^{13}C NMR spectrum of **6g**

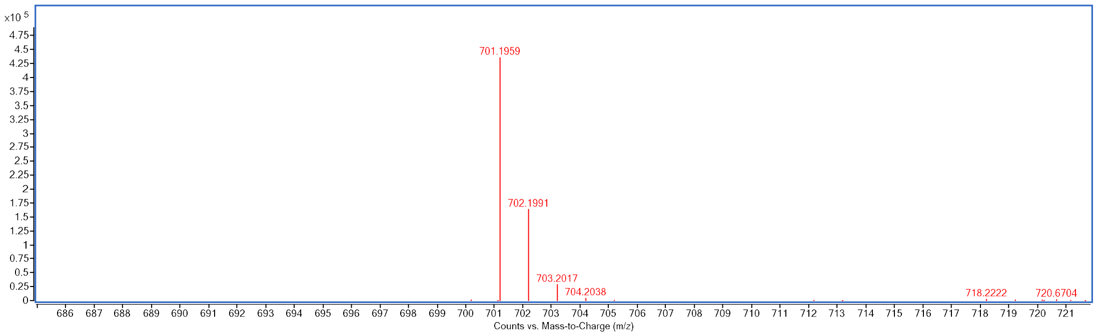

Figure S22. HRMS spectrum of **6h**

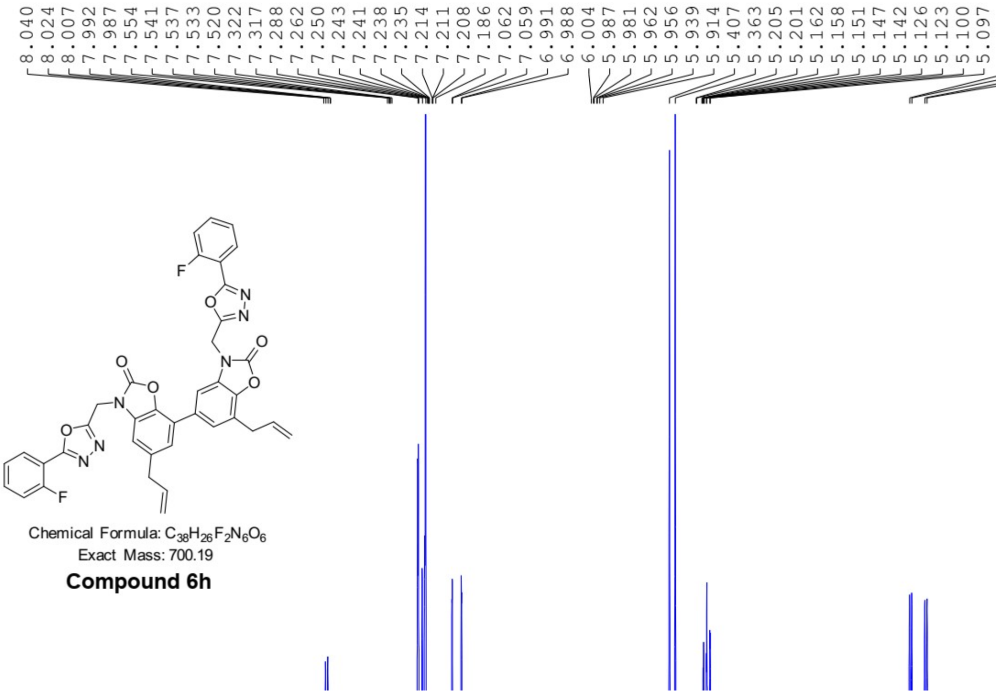

Figure S23. ^{1}H NMR spectrum of **6h**

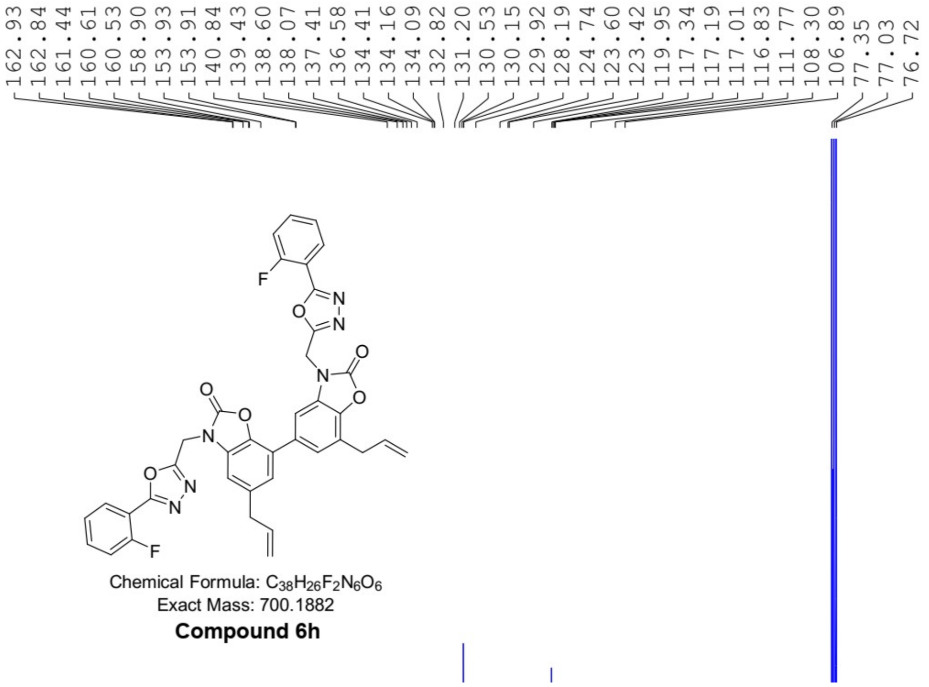

Figure S24. ^{13}C NMR spectrum of **6h**

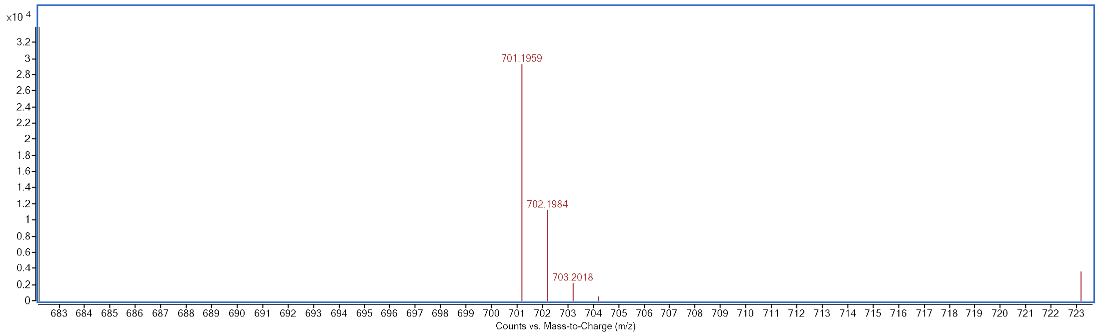

Figure S25. HRMS spectrum of **6i**

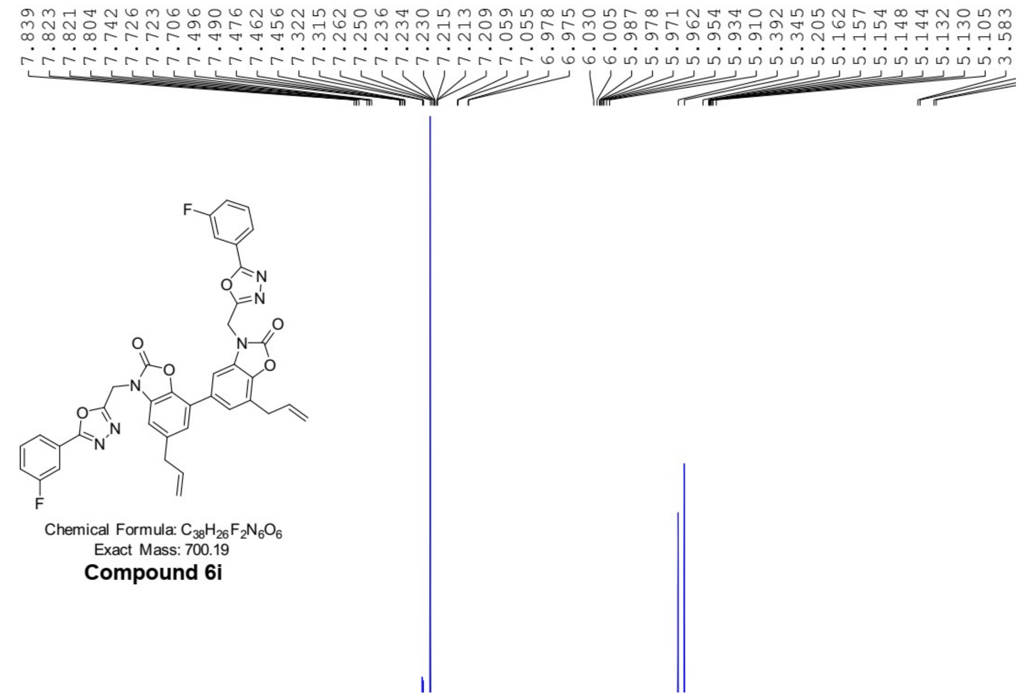

Figure S26. ^{1}H NMR spectrum of **6i**

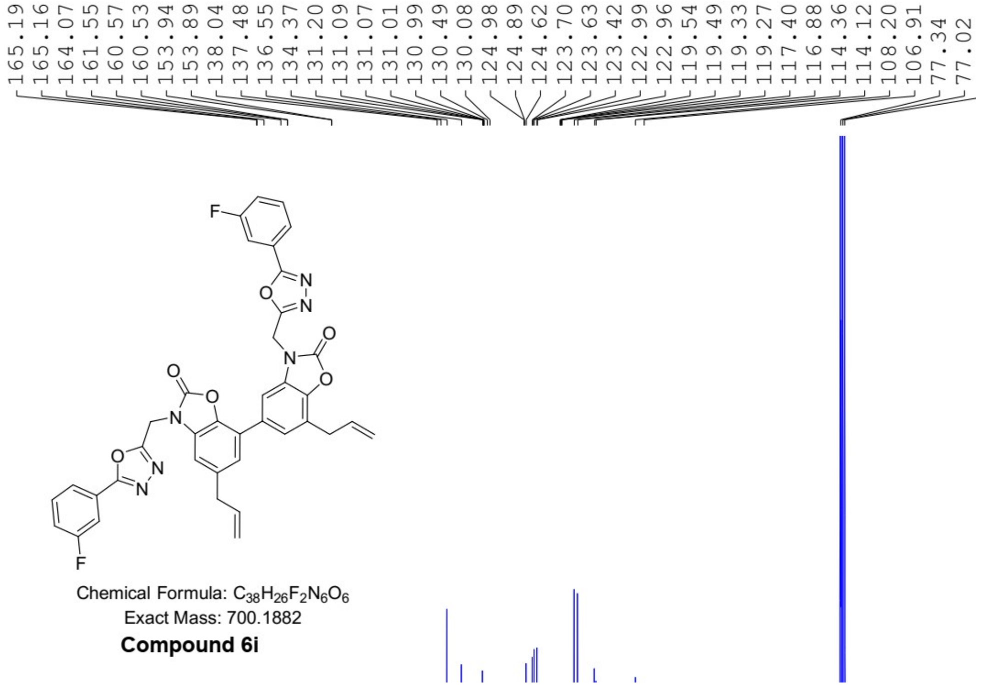

Figure S27. ^{13}C NMR spectrum of **6i**

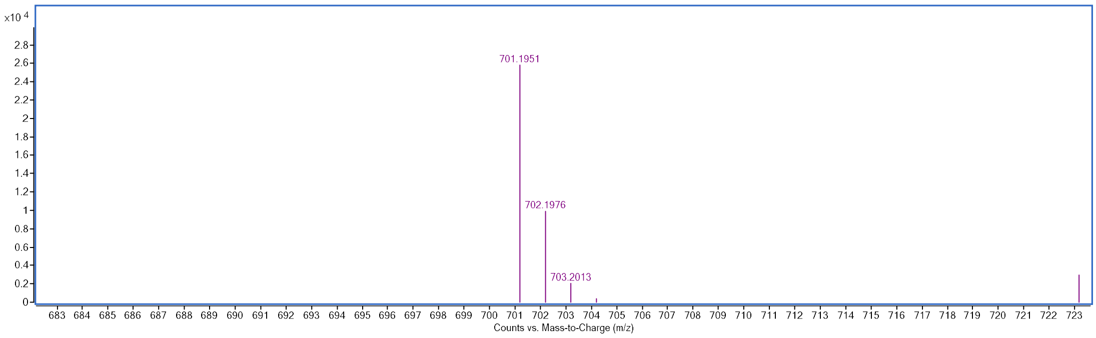

Figure S28. HRMS spectrum of **6j**

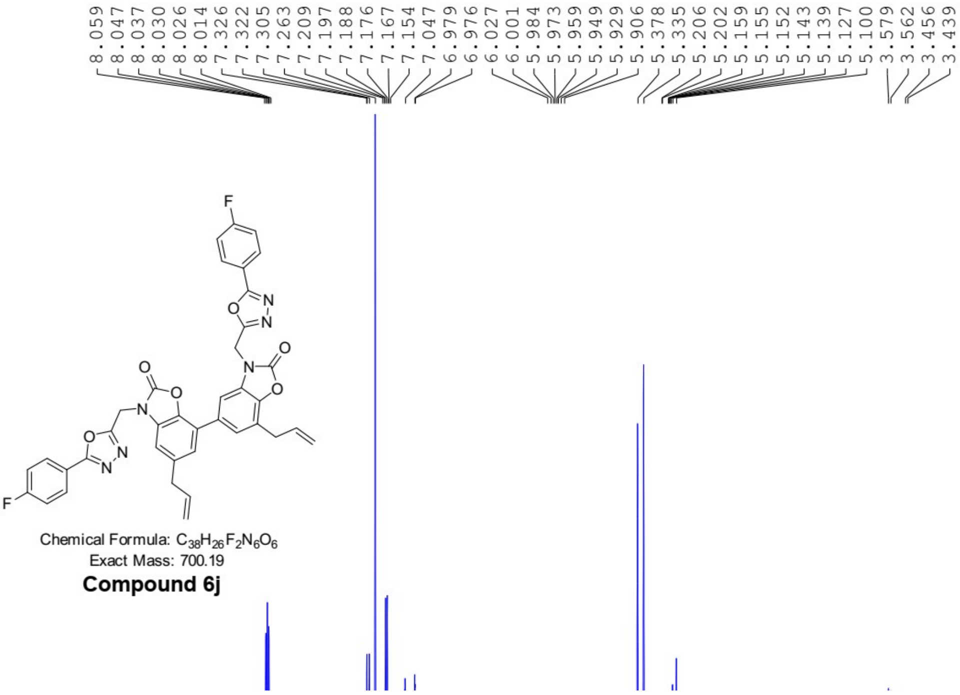

Figure S29. ^{1}H NMR spectrum of **6j**

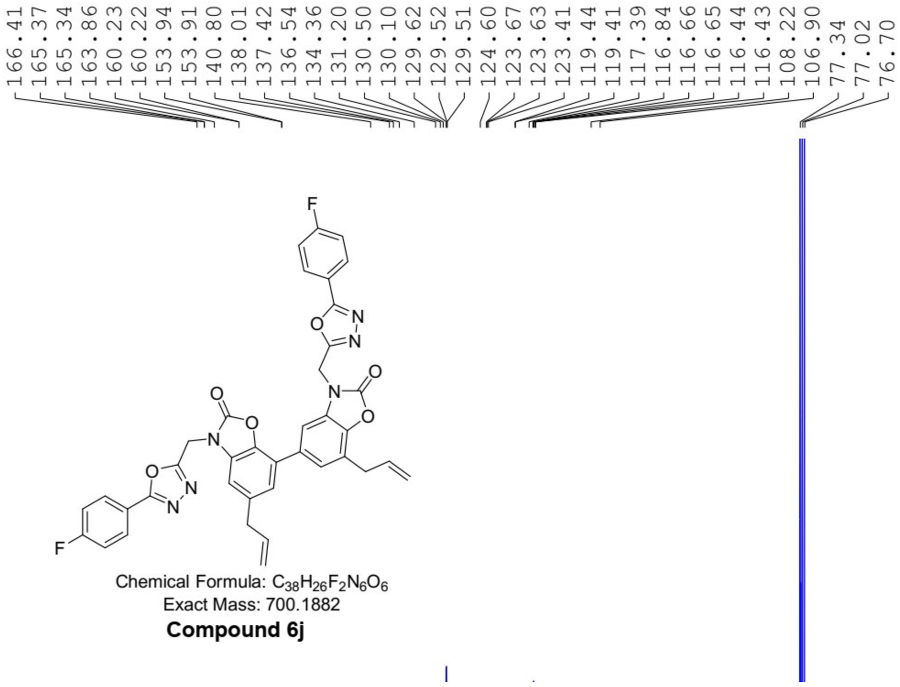

Figure S30. ^{13}C NMR spectrum of **6j**

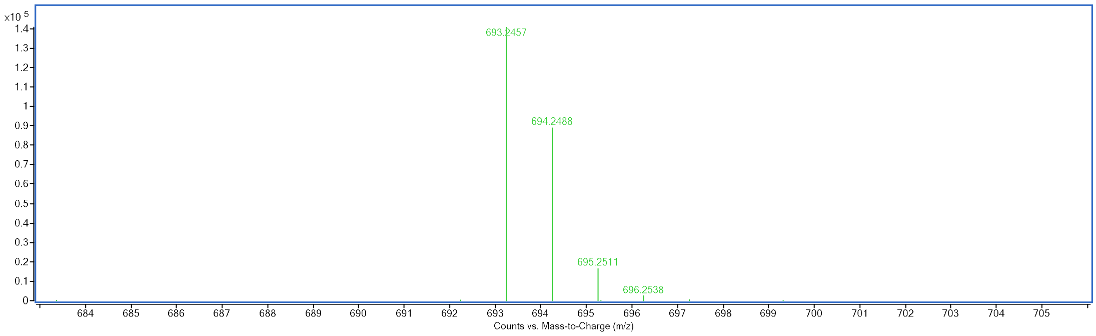

Figure S31. HRMS spectrum of **6k**

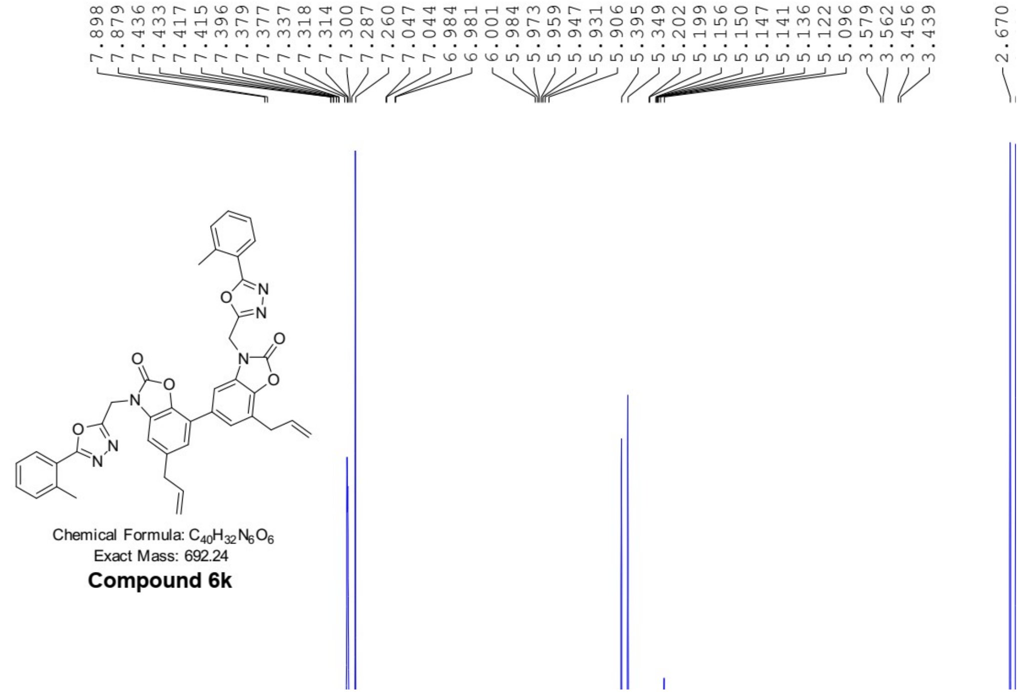

Figure S32. ^{1}H NMR spectrum of **6k**

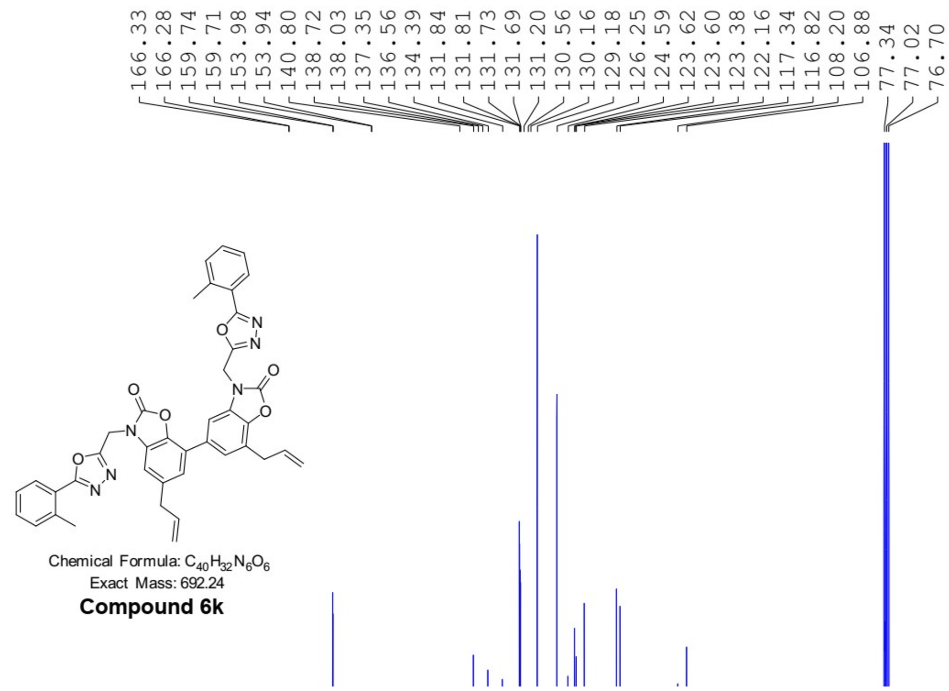

Figure S33. ^{13}C NMR spectrum of **6k**

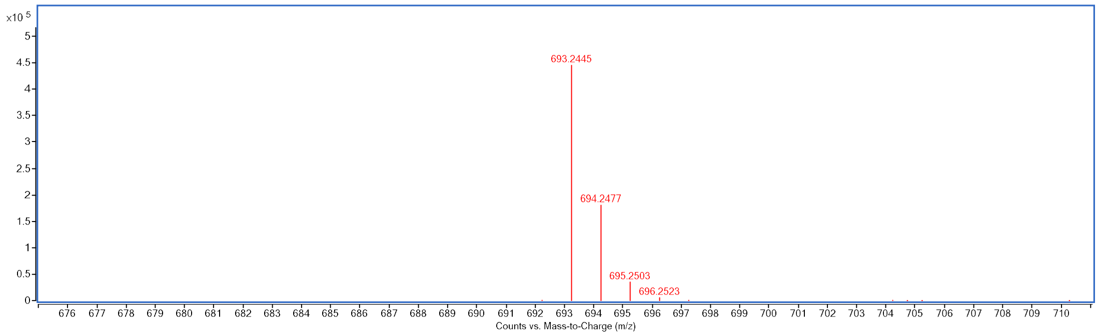

Figure S34. HRMS spectrum of **6l**

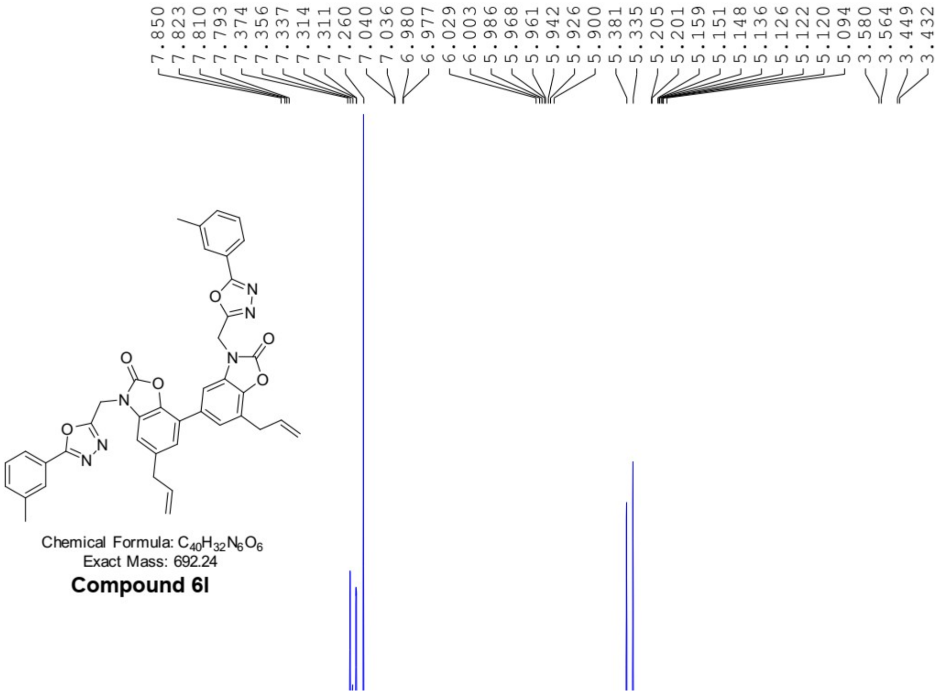

Figure S35. ^{1}H NMR spectrum of **6l**

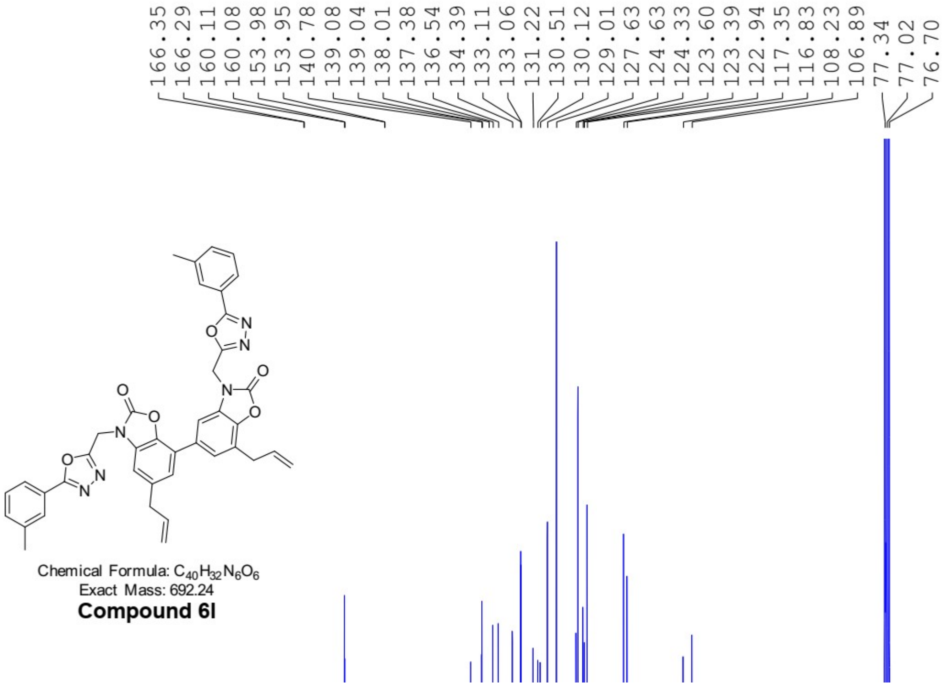

Figure S36. ^{13}C NMR spectrum of **6l**

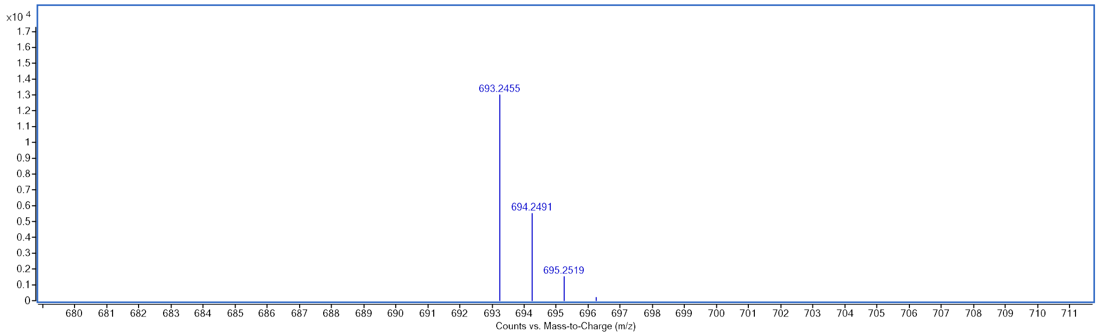

Figure S37. HRMS spectrum of **6m**

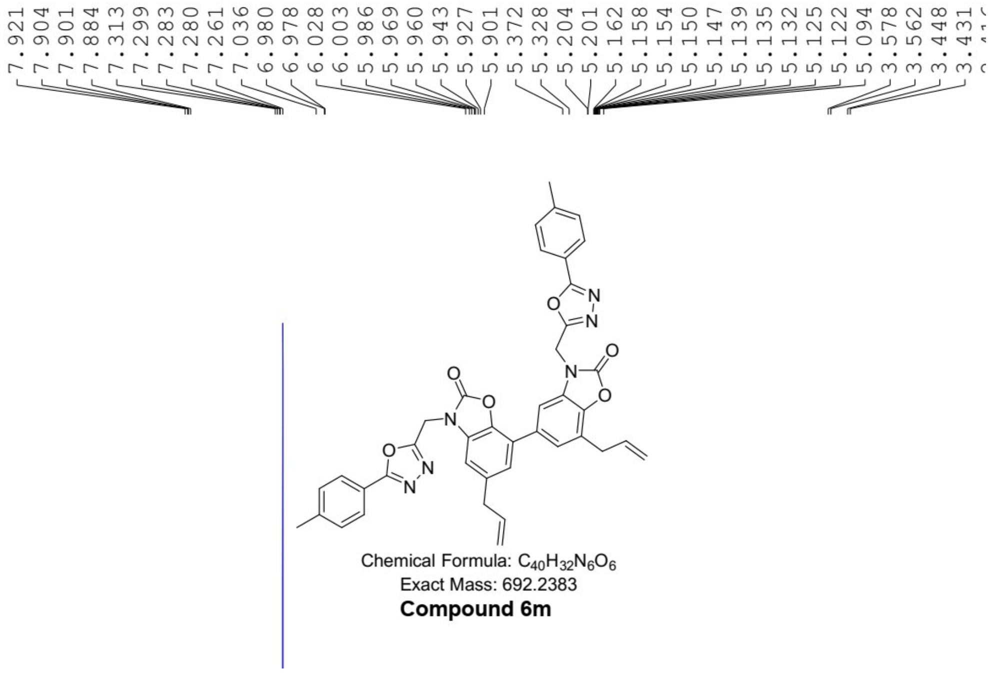

Figure S38. ^{1}H NMR spectrum of **6m**

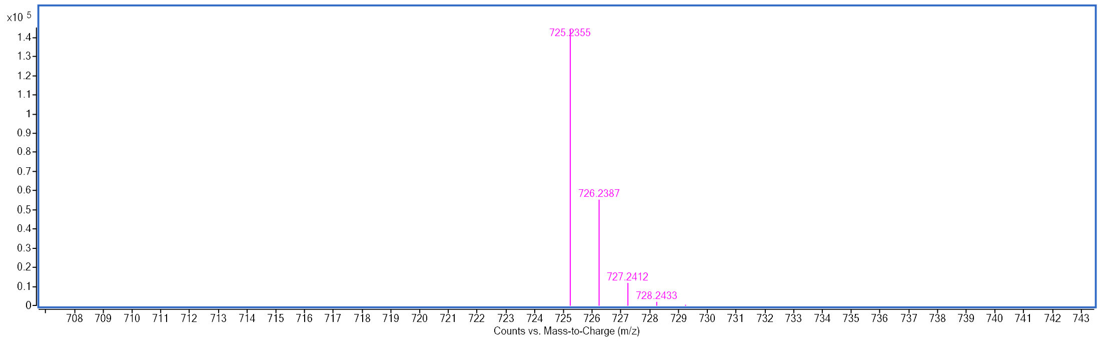

Figure S39. HRMS spectrum of **6n**

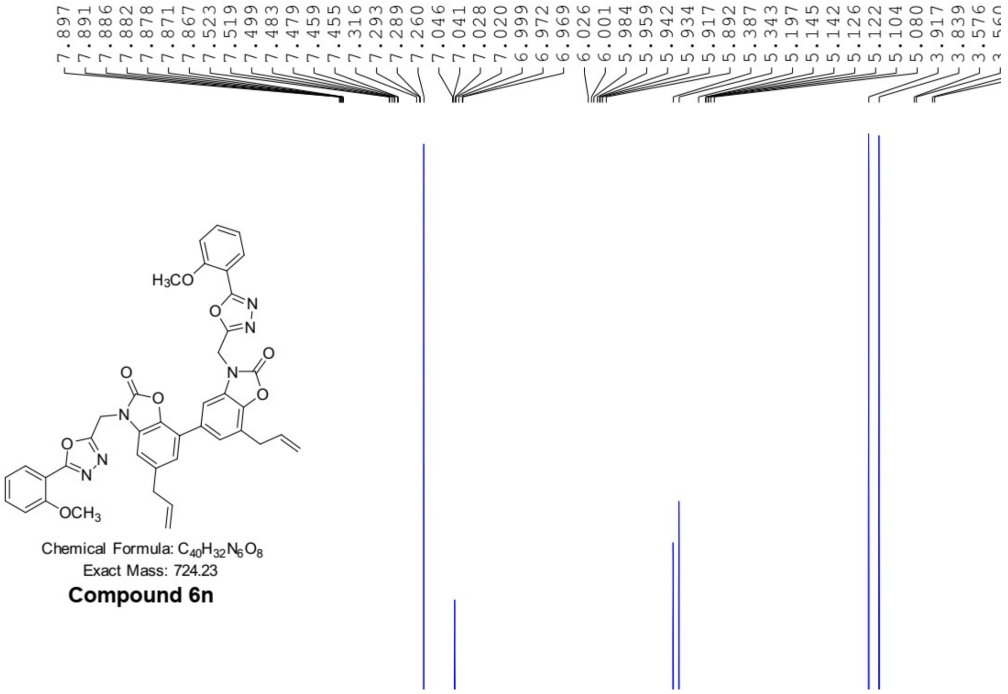

Figure S40. ^{1}H NMR spectrum of **6n**

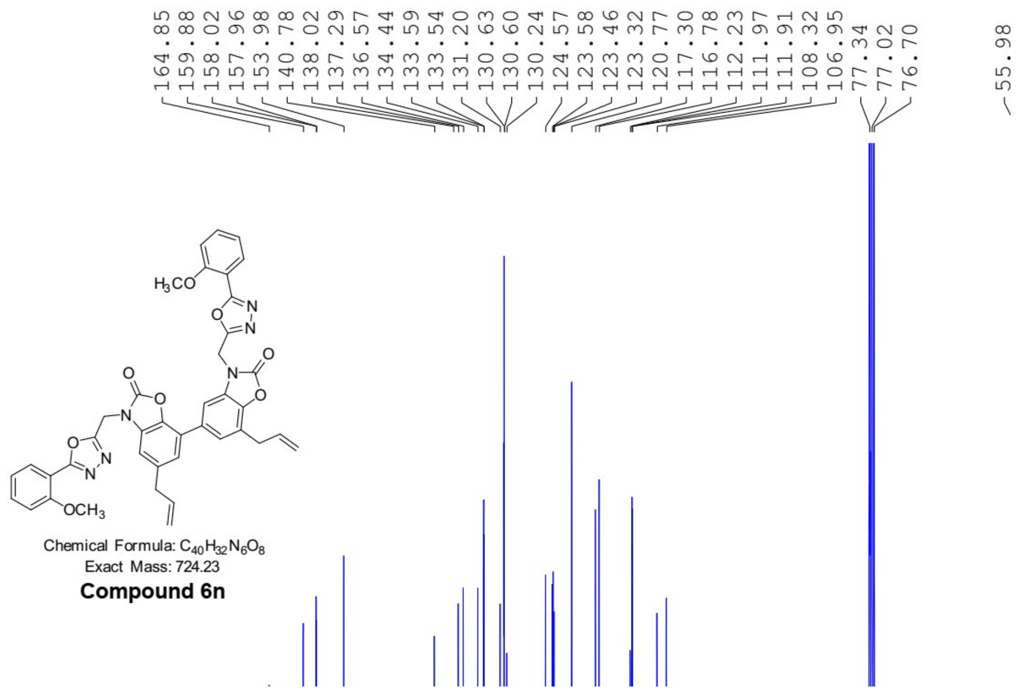

Figure S41. ^{13}C NMR spectrum of **6n**

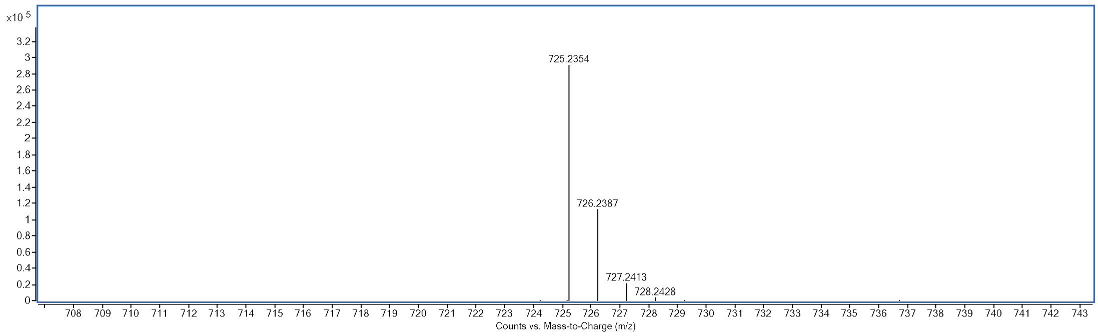

Figure S42. HRMS spectrum of **6o**

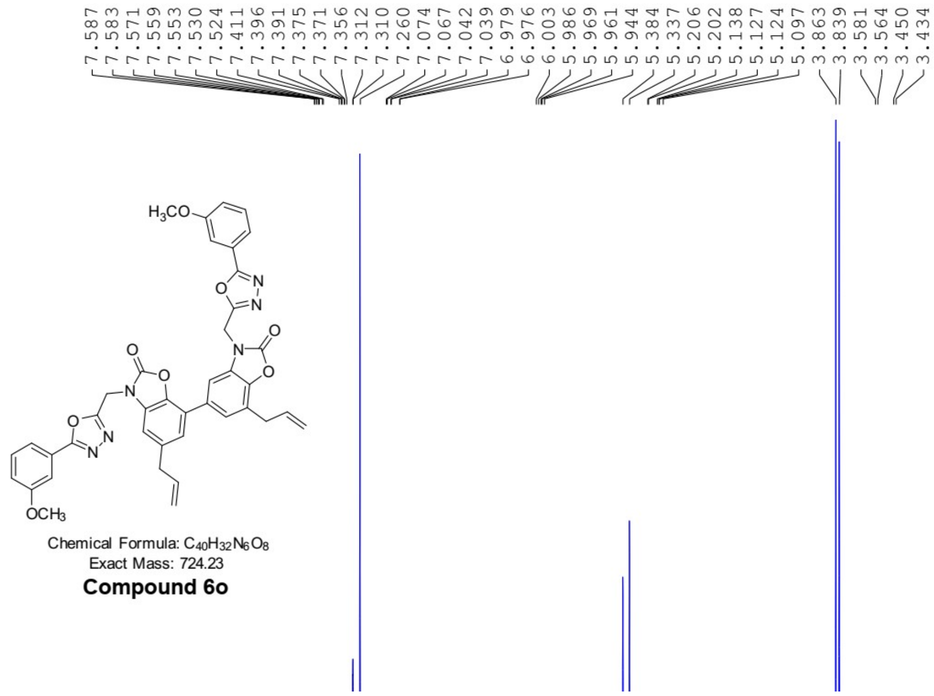

Figure S43. ^{1}H NMR spectrum of **6o**

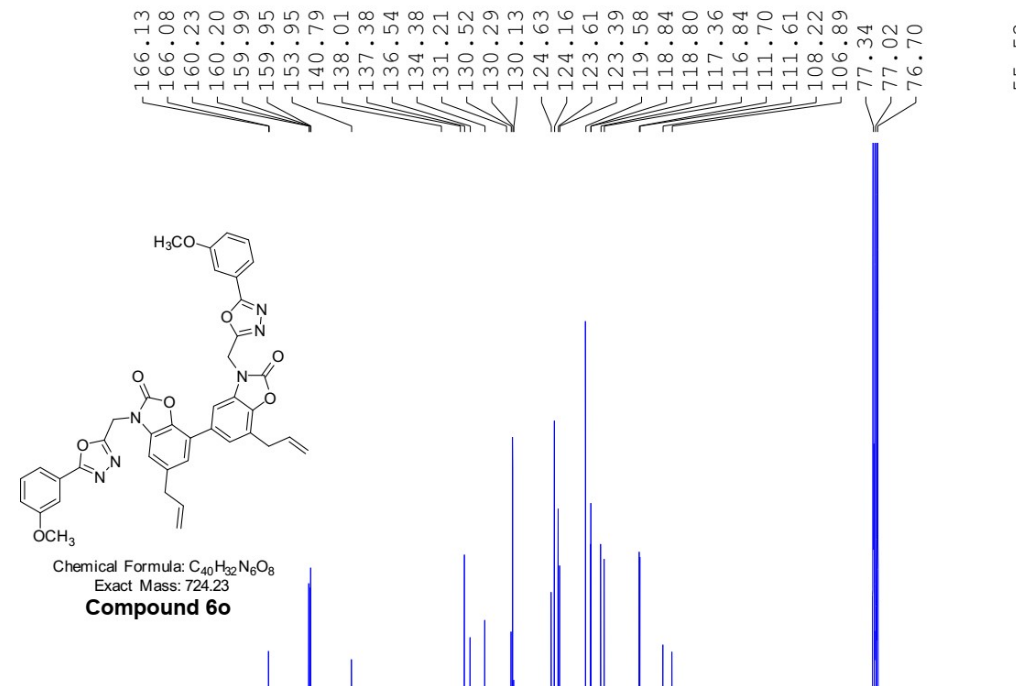

Figure S44. ^{13}C NMR spectrum of **6o**

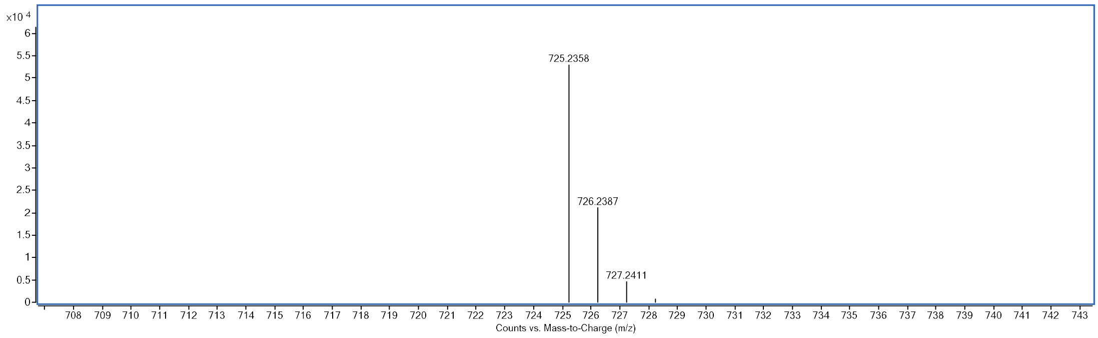

Figure S45. HRMS spectrum of **6p**

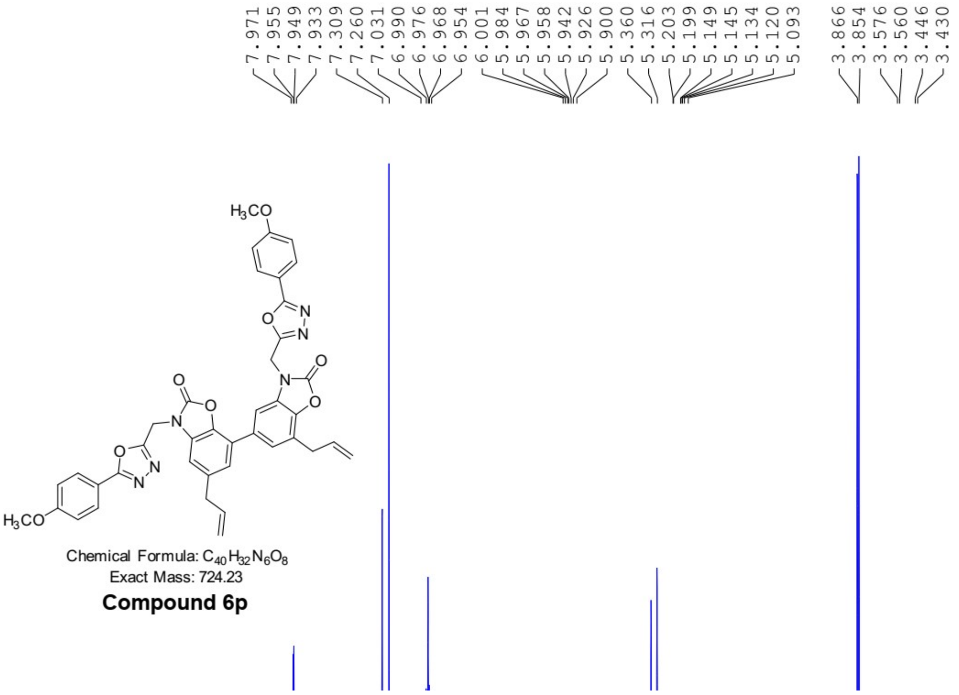

Figure S46. ^{1}H NMR spectrum of **6p**

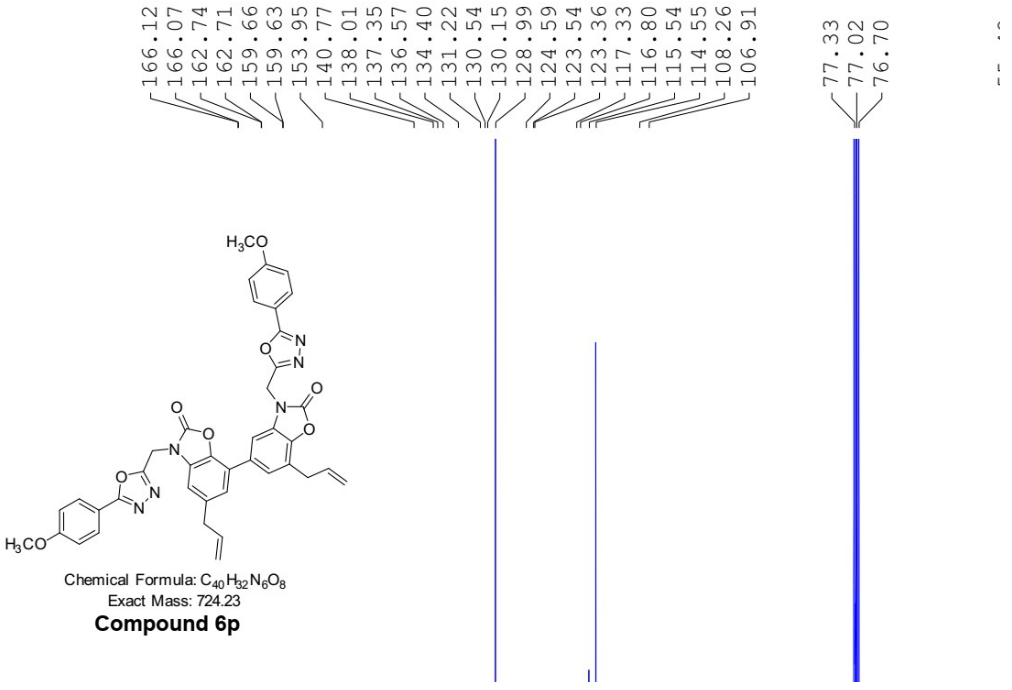

Figure S47. ^{13}C NMR spectrum of **6p**
